# Bridging In Vitro Functionality and Genomic Insights in *Lactiplantibacillus plantarum* L3 Isolated from Traditional Bulgarian Katak

**DOI:** 10.3390/ijms27167088

**Published:** 2026-08-07

**Authors:** Lili Dobreva, Nikoleta Atanasova, Petar Donchev, Ekaterina Krumova, Radoslav Abrashev, Maria Angelova, Greta Naydenova, Apostol Apostolov, Dragomir Yankov, Vladimir Tolchkov, Svetla Danova

**Affiliations:** 1 The Stephan Angeloff Institute of Microbiology, Bulgarian Academy of Sciences, 26 Acad. G. Bonchev Str., 1113 Sofia, Bulgaria; ldobreva@microbio.bas.bg (L.D.); nikoletaatanasova21@gmail.com (N.A.); petardonchev@microbio.bas.bg (P.D.); e_krumova@microbio.bas.bg (E.K.); rabrashev@microbio.bas.bg (R.A.); mariange@microbio.bas.bg (M.A.); 2 Center of Competence “Immunopathogen”, Yanko Sakuzov Blvd 26, 1504 Sofia, Bulgaria; tolchkov@ncipd.org; 3 Institute of Chemical Engineering, Bulgarian Academy of Sciences, 103 Acad. G. Bontchev Str., 1113 Sofia, Bulgaria; greta_naydenova@abv.bg (G.N.); a_apostolov@iche.bas.bg (A.A.); yanpe@bas.bg (D.Y.); 4 National Center of Infectious and Parasitic Diseases, Yanko Sakuzov Blvd 26, 1504 Sofia, Bulgaria

**Keywords:** katak, whole-genome sequencing, *Lactiplantibacillus plantarum*, candidate probiotic

## Abstract

Katak, a traditional Bulgarian fermented dairy product distinguished by its unique sensory characteristics and extended shelf life, represents a valuable source of autochthonous microorganisms whose functional and genomic properties remain insufficiently explored. In the present study, strain L3, isolated from katak, was comprehensively characterized by a polyphasic approach integrating classical microbiological methods, molecular identification, and whole-genome sequencing. Functional assessment revealed a broad spectrum of antimicrobial activity against pathogenic bacteria and food-associated fungi, accompanied by pronounced radical-scavenging and antioxidant capacities. The strain also exhibited high transit tolerance in simulated gut passage and a favorable antibiotic susceptibility profile, indicating its potential as a probiotic candidate. Whole-genome sequencing confirmed the taxonomic affiliation of the isolate as *Lactiplantibacillus plantarum* and enabled the identification of genetic determinants associated with stress response, environmental adaptation, oxidative stress protection, biofilm formation, and antimicrobial activity. The genomic analysis complemented the in vitro findings by identifying genetic determinants consistent with the observed phenotypic traits, supporting the genetic potential of strain L3 for probiotic-associated functions. The integration of phenotypic and genomic evidence allowed a comprehensive evaluation of *L. plantarum* L3 and demonstrated that its probiotic-related properties are underpinned by a diverse repertoire of functional genes. These findings identify L3 as a promising candidate for further biotechnological/probiotic assessment.

## 1. Introduction

Traditional fermented foods are increasingly recognized as valuable sources of indigenous lactic acid bacteria (LAB), enabling the isolation of novel strains with probiotic and technological potential [1,2,3,4]. The complex microbial communities naturally present in these products contribute not only to the development of their characteristic sensory properties but also to product preservation and safety. Consequently, the isolation and characterization of autochthonous LAB from traditional fermented foods have attracted considerable scientific interest in recent years.

Bulgaria possesses a rich heritage of fermented dairy products, including the globally recognized Bulgarian yogurt, as well as numerous traditional products—white brined cheese, yellow cheeses, and katak. Despite their long history of production and safe consumption, they remain insufficiently studied. These products are characterized by unique microbial ecosystems shaped by artisanal manufacturing traditions, local raw materials, and regional environmental conditions [5,6,7]. Recent studies have highlighted traditional plant- and milk-based fermented products as valuable reservoirs of LAB with promising technological and health-promoting properties [8].

Katak represents a unique Bulgarian fermented dairy food that has received relatively limited scientific attention. It has a cream cheese-like consistency and a creamy, yogurt-like texture and is traditionally produced without the addition of industrial starter cultures [9]. Its distinctive sensory characteristics, including its texture, aroma, mildly acidic flavor, and extended shelf life of up to one year, are closely associated with its indigenous microbiota, particularly LAB, which actively participate in the fermentation process and contribute to product stability [9]. Previous studies have demonstrated that LAB isolated from Bulgarian white brined cheeses [5] and katak [6], like other reports, exhibit diverse functional properties of both probiotic and technological relevance. Despite its significance as Bulgarian cultural heritage, katak remains largely unexplored from a microbiological and functional perspective, particularly regarding the diversity and characteristics of its indigenous LAB communities. Dobreva (2023, PhD thesis) identified *Lactiplantibacillus plantarum* as non-starter microbiota of this product [10]. Among the LAB commonly isolated from fermented foods, *L. plantarum* is one of the most extensively studied and industrially relevant species. Owing to its remarkable metabolic versatility and adaptability, this species is widely distributed in fermented foods of dairy, plant, and meat origin, as well as in the gastrointestinal tract of humans and animals [5,6,8,11]. In particular, *L. plantarum* was identified as the predominant species, representing 47% of the isolated LAB population, while *Lacticaseibacillus koreensis* and *Lacticaseibacillus yonginensis* were reported for the first time in Bulgarian traditional fermented foods [8].

*L. plantarum* plays a pivotal role in food fermentation through the production of organic acids, antimicrobial compounds, and other bioactive metabolites, thereby contributing to product quality, preservation, and safety. Moreover, its ability to survive gastrointestinal conditions and interact beneficially with the intestinal microbiome has established it as one of the most promising probiotic species. These characteristics have stimulated growing interest in the isolation and characterization of novel indigenous *L. plantarum* strains from traditional fermented foods as potential candidates for food biotechnology and probiotic applications.

The species also has a long history of safe use in food fermentation and has been granted Qualified Presumption of Safety (QPS) status by the European Food Safety Authority (EFSA). *L. plantarum* possesses one of the largest genomes among lactic acid bacteria, ranging from approximately 3.0 to 3.6 Mb, which contributes to its remarkable metabolic versatility, ecological flexibility, and capacity to adapt to diverse environmental niches [12]. Owing to these characteristics, *L. plantarum* has often been referred to as a “nomadic” species.

To date, public genomic databases contain several hundred *L. plantarum* genome assemblies, including more than 100 complete genomes that have served as the basis for comparative genomic analyses. Whole-genome analyses of 127 *L. plantarum* strains revealed an open pan-genome [12], reflecting considerable genomic diversity and the ongoing acquisition of genes associated with environmental adaptation, metabolic flexibility, and niche-specific functions. Consequently, newly isolated strains may harbour unique genetic determinants relevant to probiotic performance and food biotechnology applications.

This extensive genomic diversity highlights the limitations of species-level identification alone. Although two strains may share more than 99% similarity in their 16S rRNA gene sequences, they can exhibit markedly different technological, functional, and probiotic properties. Individual strains may possess unique bacteriocin biosynthetic gene clusters, genes involved in exopolysaccharide production, specialized metabolic pathways, or specific genetic adaptations to dairy environments. Therefore, strain-level genomic characterization is essential for accurately predicting functional potential and technological performance.

Given the remarkable genomic diversity of *L. plantarum* and the increasing importance of whole-genome sequencing for strain characterization, safety assessment, and functional prediction, the genomic investigation of autochthonous isolates from traditional Bulgarian fermented foods may reveal novel strains with significant probiotic and biotechnological potential.

In view of the substantial strain-level diversity within *L. plantarum* and the growing importance of genome-based characterization of food-associated microorganisms, the present study aimed to investigate the functional phenotype and genomic repertoire of the L3 strain, isolated from the traditional Bulgarian product katak. To achieve this objective, the strain was evaluated through a combination of in vitro functional assays and whole-genome sequencing analyses. Particular emphasis was placed on genetic determinants associated with environmental adaptation, stress tolerance, antimicrobial activity, technological performance, and safety.

The integration of phenotypic and genomic approaches was intended to provide a comprehensive assessment of the strain’s potential for application in food biotechnology and probiotic development.

## 2. Results

### 2.1. Isolation and Polyphasic Taxonomy Identification of L3 Strain from Katak

**Figure 1:**
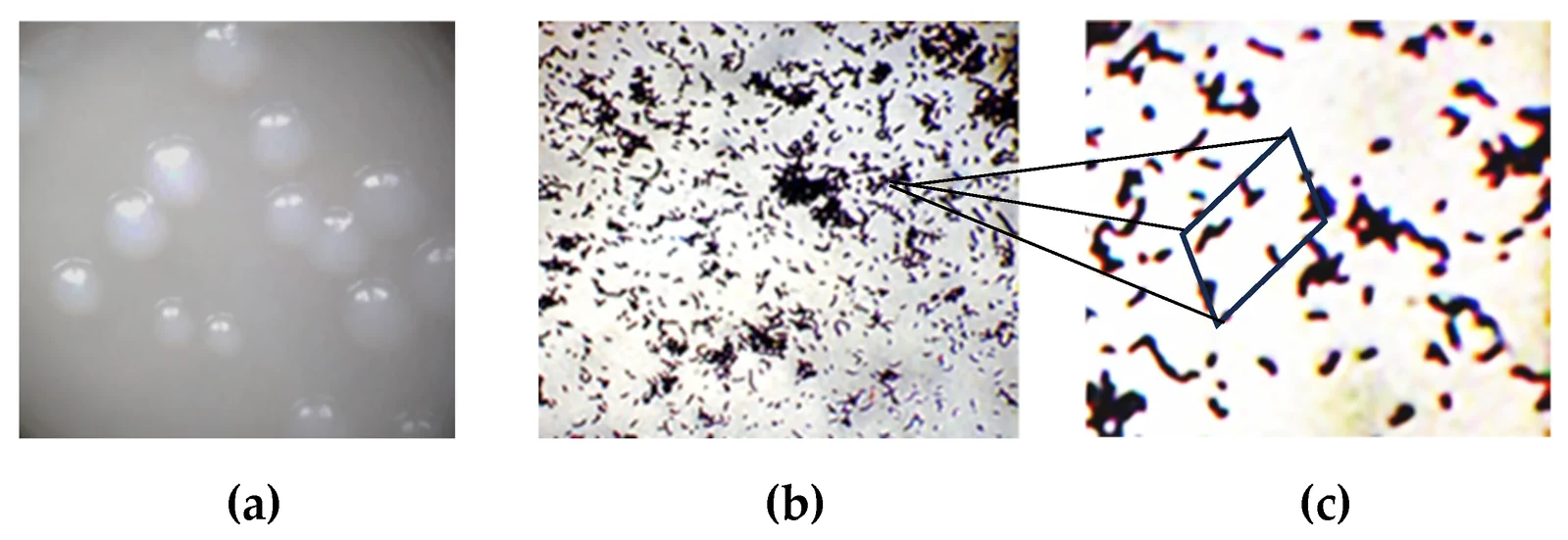
Macro- and micromorphological characteristics of the selected isolate L3 from katak: (**a**) morphology of colonies on Rogosa agar; (**b**) microscope images of L3 pure culture cells—magnification 800×; and (**c**) L3 cells—magnification 2000× (BestScope BUC4-500C S/NBS693201005904BS-LED) Trinocular LED Fluorescent Biological Microscope (Beijing Bestscope Technology Co., Ltd., Beijing, China).

Twenty-five pure cultures with morphological characteristics of LAB were successfully isolated from katak samples, collected from a small farm near the city of Lukovit, Bulgaria (43°12′ N 24°10′ E/43.200° N 24.167° E). Thus, only Gram-positive, non-motile, non-spore-forming, microaerophilic, oxidase- and catalase-negative bacteria were isolated and preserved in the internal culture collection of the Laboratory of Lactic Acid Bacteria and Probiotics, Institute of Microbiology, Bulgarian Academy of Sciences (Grant KH-36/4, National Scientific Fund). Distinct colonies developed on Rogosa agar after 48 h of incubation at 37 °C (Figure 1a). Purification and repeated subculturing in MRS agar and MRS broth under anaerobic conditions were performed, and pure cultures of isolates (L3, L9, and L12) with similar colony morphology were obtained (Figure 1a). Phenotypic characterization revealed that the selected isolates were non-motile, catalase-negative, Gram-positive bacteria with rod-shaped morphology. Together with the other tests, the results were consistent with those typically observed in lactobacilli. The isolate L3 (Figure 1b,c), preselected for the present study, was stored in MRS broth (HiMedia, Mumbai, India), supplemented with 20% *v*/*v* glycerol, at −20 °C for further analyses.

Initial identification of isolate L3 was performed using polyphasic taxonomy, with classical morpho-physiological tests (Figure 1), followed by highly discriminative molecular analyses and full genome sequencing. The full-length 16S rRNA gene sequence classified the L3 strain in close relation with 3 species from the genus *Lactiplantibacillus*—*L. paraplantarum*, *L. plantarum* and *L. pentosus*. BLAST analysis of the 16S rRNA sequence of L3 with data from NCBI GenBank showed ~100% identity with *L. plantarum* JCM 1149 (GenBank accession number NR_115605.1), *L. pentosus* strain 124-2 (NR_029133.1) and *L. paraplantarum* DSM 10667 (NR_025447.1) (Supplementary Materials). The protocol, including multiplex PCR targeting the *recA* gene, does not allow discrimination and L3 identification. Therefore, whole-genome sequencing (WGS) was performed, and the isolate was identified as *L. plantarum* (GenBank accession number GCF_036353295.2; BioProject: PRJNA1050159; Bio Sample: SAMN38726275). To further confirm the taxonomic assignment, genome-based comparison using OrthoANI analysis was performed. The L3 genome exhibited high average nucleotide identity (ANI) values with reference genomes of *Lactiplantibacillus plantarum*, showing 99.07% and 98.04% identity with strains ASM991365v1 and WCFS1, respectively. In contrast, substantially lower ANI values were observed with *L. paraplantarum* (86.47%) and *L. pentosus* (80.41%) (Table 1). Consequently, the strain was identified as *L. plantarum* and is hereafter referred to as *L. plantarum* throughout the discussion of the in vitro experiments.

The species *L. plantarum* is among the most widespread species that have been successfully isolated from various ecological niches and are considered to have biotechnological and probiotic applications. With this aim, the in vitro spectrum of activity of the newly identified strain L3 was assessed.

### 2.2. In Vitro Evaluation of Functional Characteristics and Probiotic Potential of L. plantarum L3 Isolated from Katak

Autochthonous dairy microbiota is the subject of intensive research as bioprotective probiotics that naturally limit the invasion of undesirable bacteria and help protect the food chain from foodborne and spoilage microbes. Understanding the key role of LAB in combating pathogens is an important step in the selection of biotechnologically relevant and probiotic strains. Thus, the first step in characterizing L3 as a candidate probiotic was to assess its biological activity.

#### 2.2.1. Antibacterial Activity of Strain L3

The antibacterial assays were evaluated against the Gram-positive strains *Staphylococcus aureus* V00037 and *Streptococcus mutans* DSMZ 20523, as well as the Gram-negative strains *Escherichia coli* ATCC 25922 and HB101 and *Pseudomonas aeruginosa* PAO1. To simulate different conditions within the food chain, *L. plantarum* L3 was cultured in MRS broth at 37 °C, in reconstituted 10% *w*/*v* skimmed cow milk (Serva), and in freshly prepared soy milk. The agar diffusion method was used for initial estimation of the inhibitory potential of cell-free supernatants (CFS) from L3 cultures in exponential (24 h) and stationary phase (48 h) grown in MRS broth (pH 6.5), as well as whey fractions from fermented skimmed and soy milk (Table 2). The antibiotics erythromycin (Bulbio, 15 μg/disc), chloramphenicol (Bulbio, 30 μg/disc), and ciprofloxacin (Bulbio, 5 μg/disc) were used as controls. The neutralized CFS (nCFS) showed higher antibacterial activity against *E. coli* HB101 than the ciprofloxacin control. The activity of proteinase K-treated CFS was lower than that of the acidic CFS (aCFS), suggesting the presence of antibacterial metabolites of a different nature. A 3.3% acetic acid solution (pH 4.75) was used as a control and resulted in smaller inhibition zones compared with those observed for aCFS and the treated CFS samples (Table 2).

#### 2.2.2. Antifungal Activity of *L. plantarum* L3

**Figure 2:**
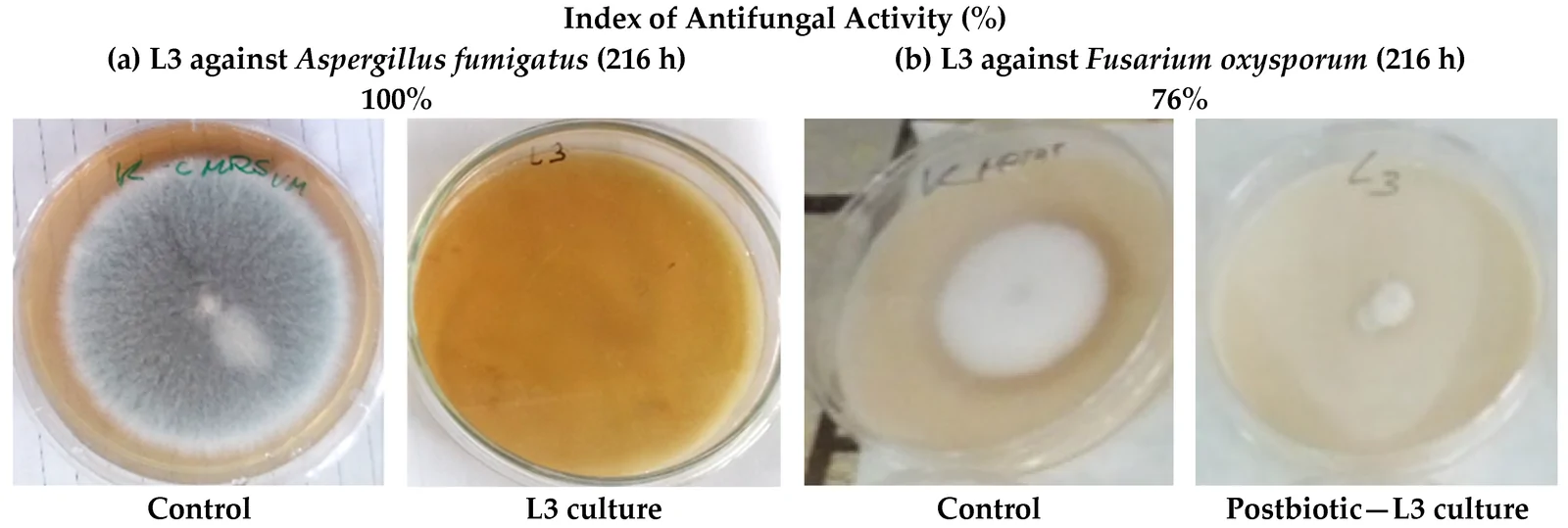
In vitro assessment of antifungal activity of exponential L3 cultures against *A. fumigatus* and *F. oxysporum* by modified agar diffusion method: (**a**) two-layer assay MRS + PDA agar and (**b**) one-layer assay in PDA agar. Nystatin (10 mg/mL) was used as a positive control. Sterile MRS broth was used as a negative control.

Lactic acid bacteria (LAB), and particularly *L. plantarum*, are considered promising biopreservatives for controlling mold in food and agriculture [13]. Various variants of the agar-diffusion method have been adapted to estimate the bioprotective potential of *L. plantarum* L3. The strain L3 showed a promising spectrum of antifungal activity against *Aspergillus fumigatus* and *Fusarium oxysporum*, with an Index of Antifungal Activity (IAA) of 100% and 76%, respectively (Figure 2).

Antifungal activity was determined by different variants of the agar-diffusion method: (i) two-layer assay (MRS + PDA agar) (Figure 2a), which allowed the L3 culture to grow for 24 h in optimal LAB agar medium and to produce active metabolites; (ii) overlayed PDA agar is an optimal medium for fungal growth media explored for 216 h (Figure 2b). The control was supplemented with 10% *v*/*v* of MRS broth, without L3 inoculum. In the one-layer variant, only PDA agar was used, and the postbiotic—24 h L3 cultures (cells + CFS) were inoculated at 10% *v*/*v*.

**Figure 3:**
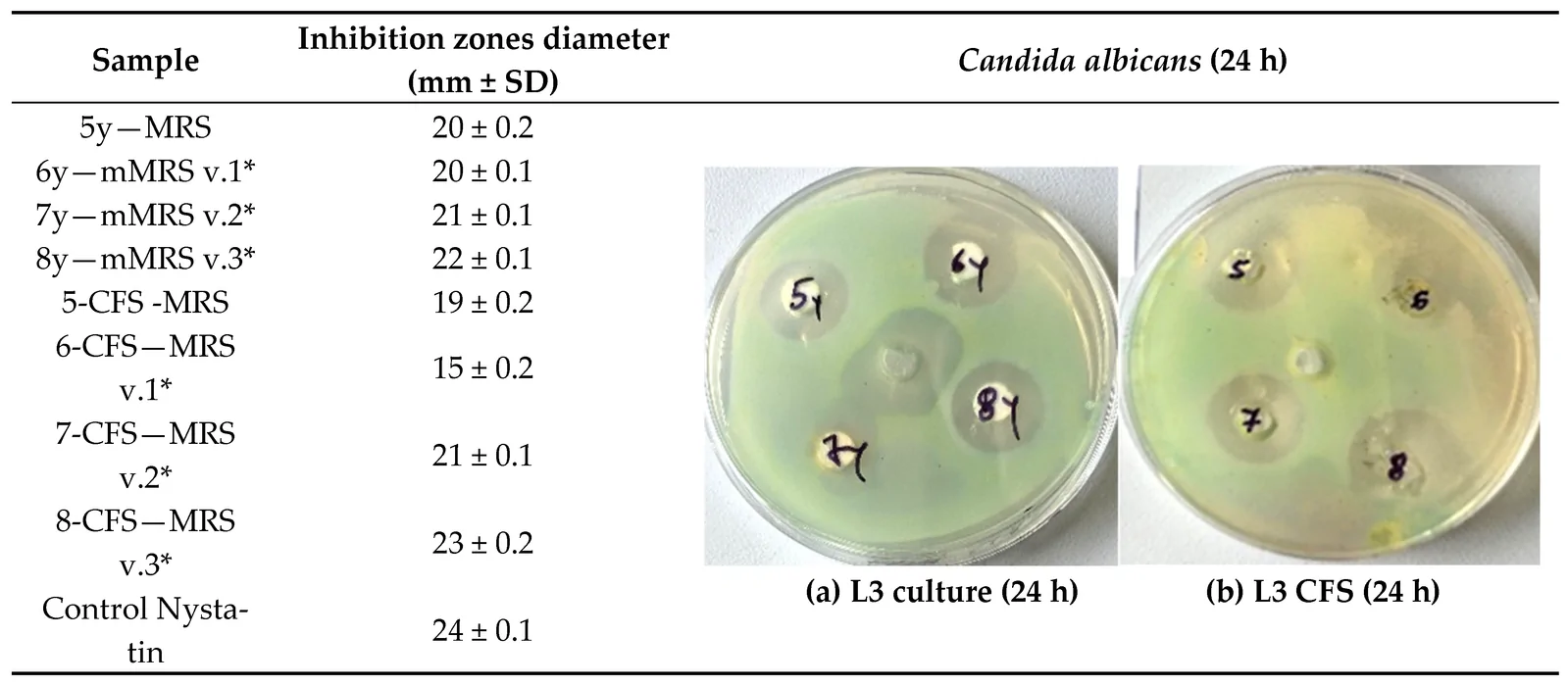
Anti-*Candida* activity (presented as diameter of zone of inhibition) of *L. plantarum* L3 culture (**a**) and of metabiotics produced during fermentation in variants of MRS broth for 24 h at 37 °C (**b**). * Legend—media for *L. plantarum* L3 cultivation: MRS (HiMedia, Mumbai, India); mMRS v.1—with lactose 20% *w*/*v*; mMRS v.2—MRS (HiVeg, India) and mMRS v.3—with glucose + soya peptone.

**Figure 4:**
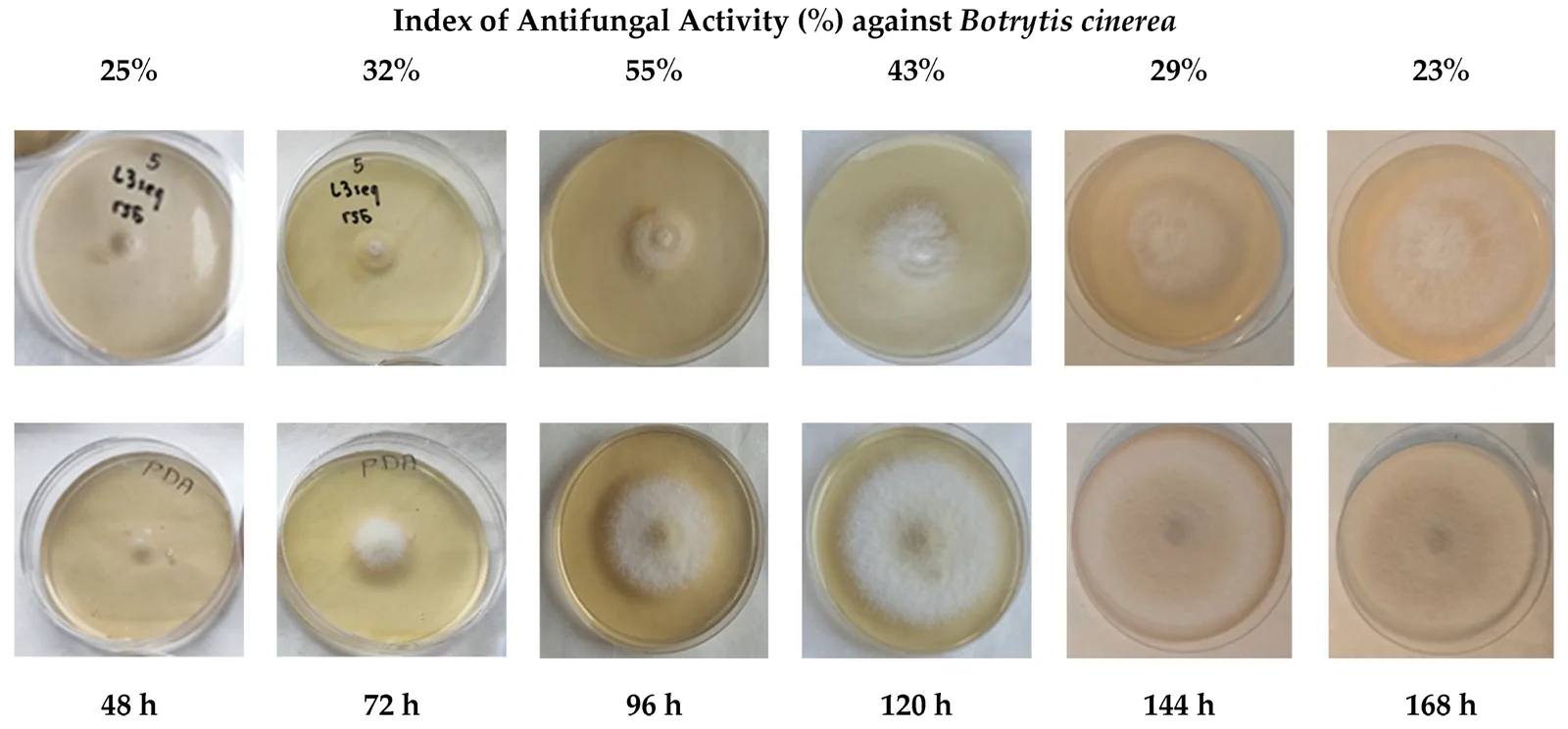
In vitro assessment of antifungal potential of filtered CFS (from 24 h culture in modified MRS broth), supplemented in PDA (10% *v*/*v*) against *Botrytis cinerea* NBIMCC 120. Results are presented as Index of Antifungal Activity (IAA) according to the formula: IAA (%) = (1 − Dsample/Dcontrol) × 100.

Cell-free supernatants collected during the cultivation of *L. plantarum* L3 at 37 °C in MRS medium (HiMedia, Mumbai, India) supplemented with glucose and MRS variants also successfully inhibited *Candida albicans* (Figure 3). Anti-*Candida* activity was measured in Sabouraud agar using the well agar-diffusion method. Both the exponential L3 culture (24 h) in MRS and the filtered cell-free supernatants showed comparable antimicrobial activity, as indicated by similar inhibition zone diameters (Figure 3). The same effect was observed after application of 48-h cultures. Meanwhile, filtered CFS from different cultivation media variants, added at 10% *v*/*v* to PDA, showed an IAA (Index of Antifungal Activity) ranging from 25% to 55% against *Botrytis cinerea* during a 144 h in vitro assay (Figure 4).

#### 2.2.3. Metabolomic Analysis of Cell-Free Supernatants Obtained During the Fermentation of *L. plantarum* L3 in MRS Broth

Given the pronounced antifungal potential exhibited during the fermentation of L3 strains, further investigation was undertaken to identify the metabolites potentially responsible for their antagonistic activity. For this purpose, gas chromatography–mass spectrometry (GC–MS) was employed to characterize the compounds present in the analyzed lyophilized cell-free supernatants (Table 3). The widely reported antimicrobials β-phenyllactic, lactic, and butyric acids were recorded by GS-MS analyses.

#### 2.2.4. Antioxidant Activity of Strain L3 from Katak

Since lactobacilli are widely involved in food fermentation, their antioxidant properties are of considerable scientific interest.

**Figure 5:**
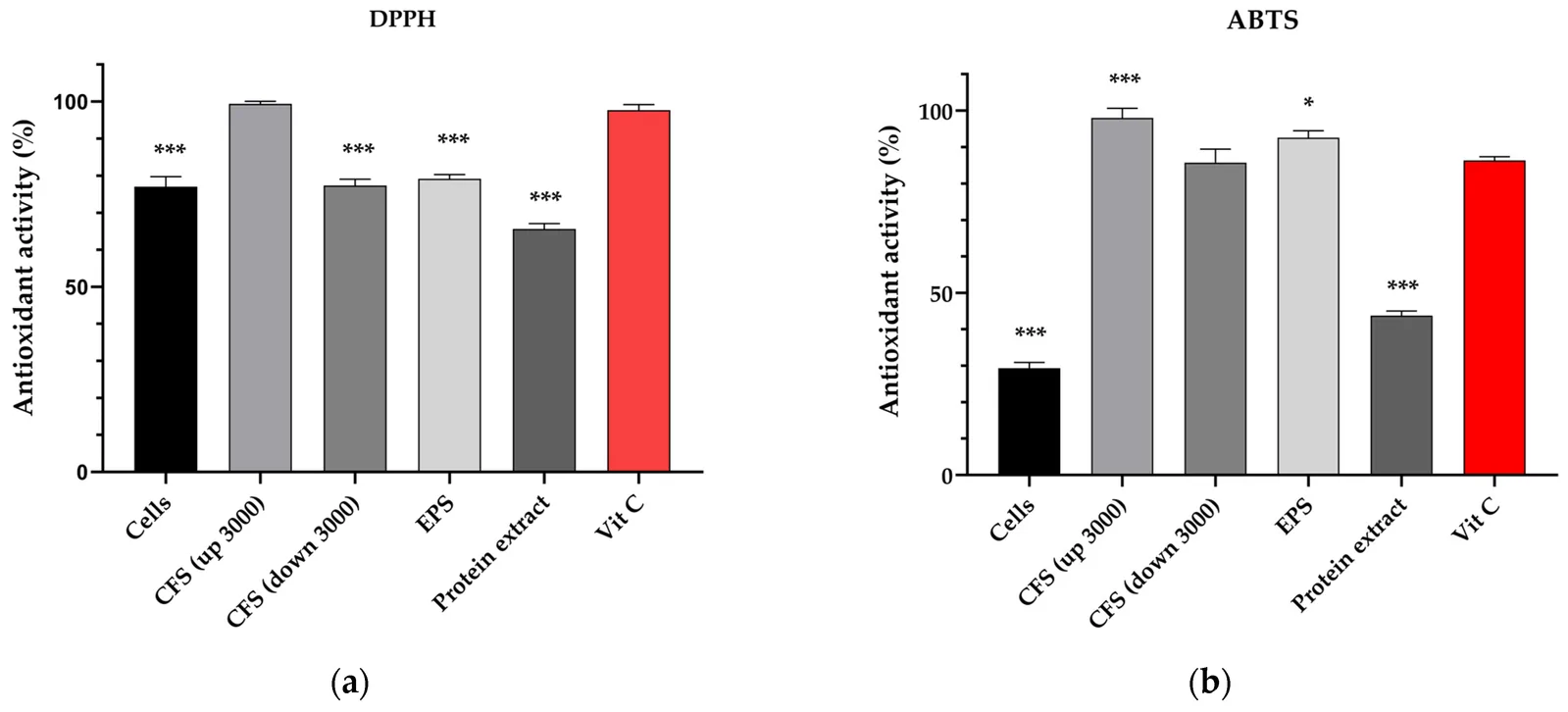
Antioxidant activity of *L. plantarum* L3 cells and their metabolites: (**a**) DPPH and (**b**) ABTS methods. Data are represented as mean ± SD (n = 3). Ascorbic acid (0.1 g/mL) was used as a positive control. * *p* < 0.05; *** *p* < 0.001 (ANOVA). The red bars represent the positive control used—vitamin C (SOPHARMA, Pharmacuticals, Bulgaria).

The antioxidant activities of the tested supernatants and live *L. plantarum* L3 cells, assessed using the DPPH and ABTS assays, are summarized in Figure 5. The DPPH radical scavenging activity is based on the ability of antioxidants to donate hydrogen atoms. Subsequently, ultrafiltration of the active L3 (≤3000 Da) resulted in the highest antioxidant activity, comparable to that of the control vitamin C in both assays (Figure 5). The radical scavenging activity of acidic L3 CFS ranged between 77 and 99% by both methods, whereas higher activity was observed for whole cells in the DPPH assay (77%) (Figure 5b). This assay suggests that high-molecular-weight compounds are major contributors to the antioxidant potential of this strain. The L3 exopolysaccharide (EPS-protein), obtained by ethanol extraction (1 CFS: 4 EtOH), also demonstrated considerable antioxidant activity, with DPPH and ABTS-scavenging activities ranging between approximately 79 and 92%, respectively.

#### 2.2.5. Survival Under Simulated Gastrointestinal Conditions in an In Vitro Model and Biofilm-Formation Potential of *L. plantarum* L3

**Figure 6:**
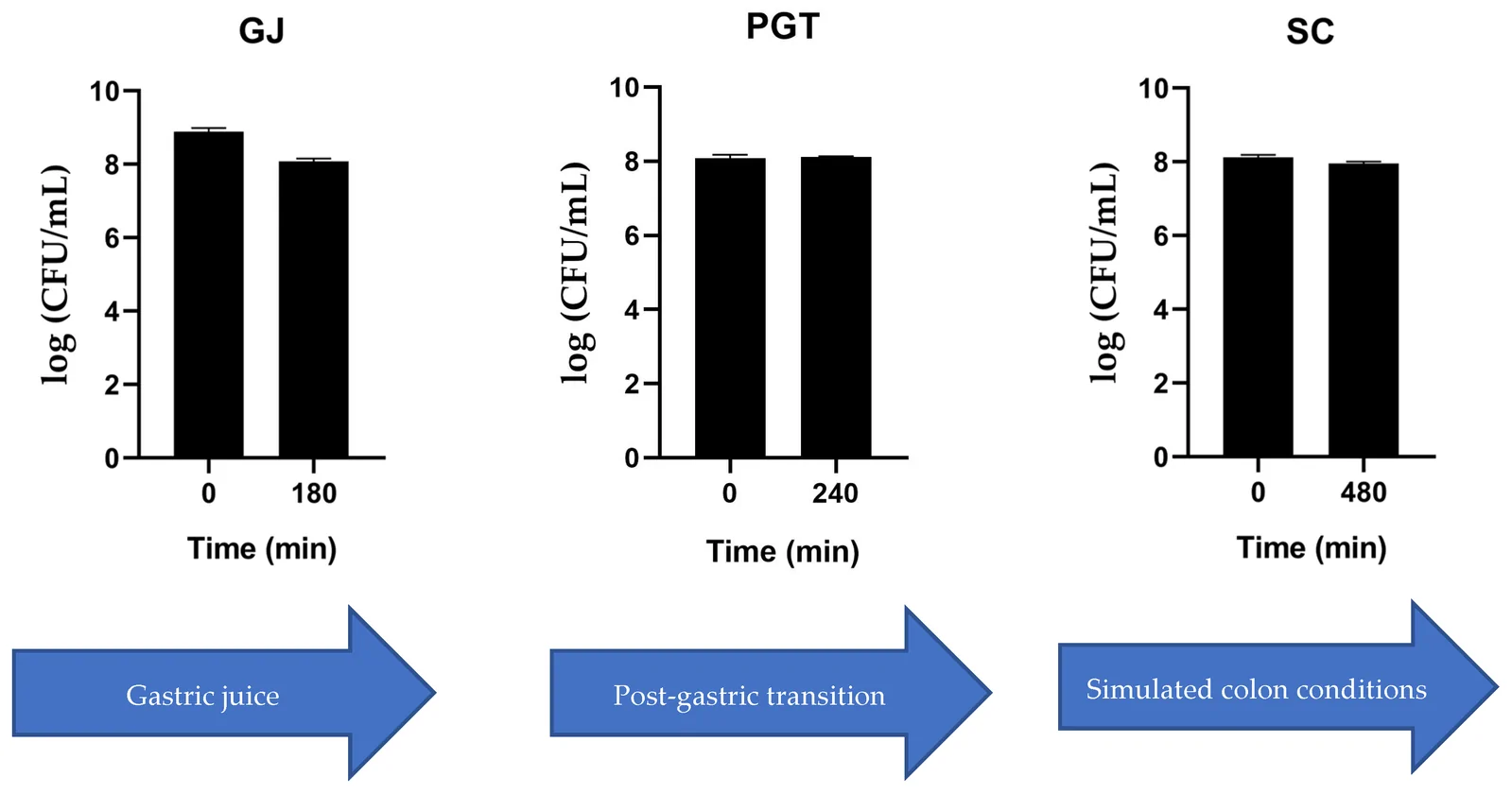
Viability of *L. plantarum* L3 in dynamic model of passage in different parts of gastrointestinal tract according to modified methods of Charteris et al. (1998) [14].

The survival of strain *L. plantarum* L3 under simulated gastrointestinal conditions was evaluated using a sequential in vitro model with a modification of Charteris et al. (1998) [14]. The microplate model system involves sequential exposure to simulated gastric juice (GJ), followed by cultivation in modified intestinal juice containing bile salts and pancreatin at pH 6.0 (PGT) as a model of the proximal part—jejunum, and simulated distal part—pH 8.0 in the presence of mucin (SC) (Figure 6). Following 180 min of incubation in simulated gastric juice, strain L3 exhibited a reduction of approximately 0.8 log CFU/m (Figure 6). During the subsequent intestinal phase, no reduction in viability was observed, and bacterial counts remained stable (8.12 log CFU/mL after 240 min). After further incubation under distal intestinal conditions for 480 min, only a slight decrease in viability was detected, with cell counts reaching 7.945 log CFU/mL. Overall, the greatest reduction in the viability of strain L3 occurred during the gastric phase at low pH.

**Figure 7:**
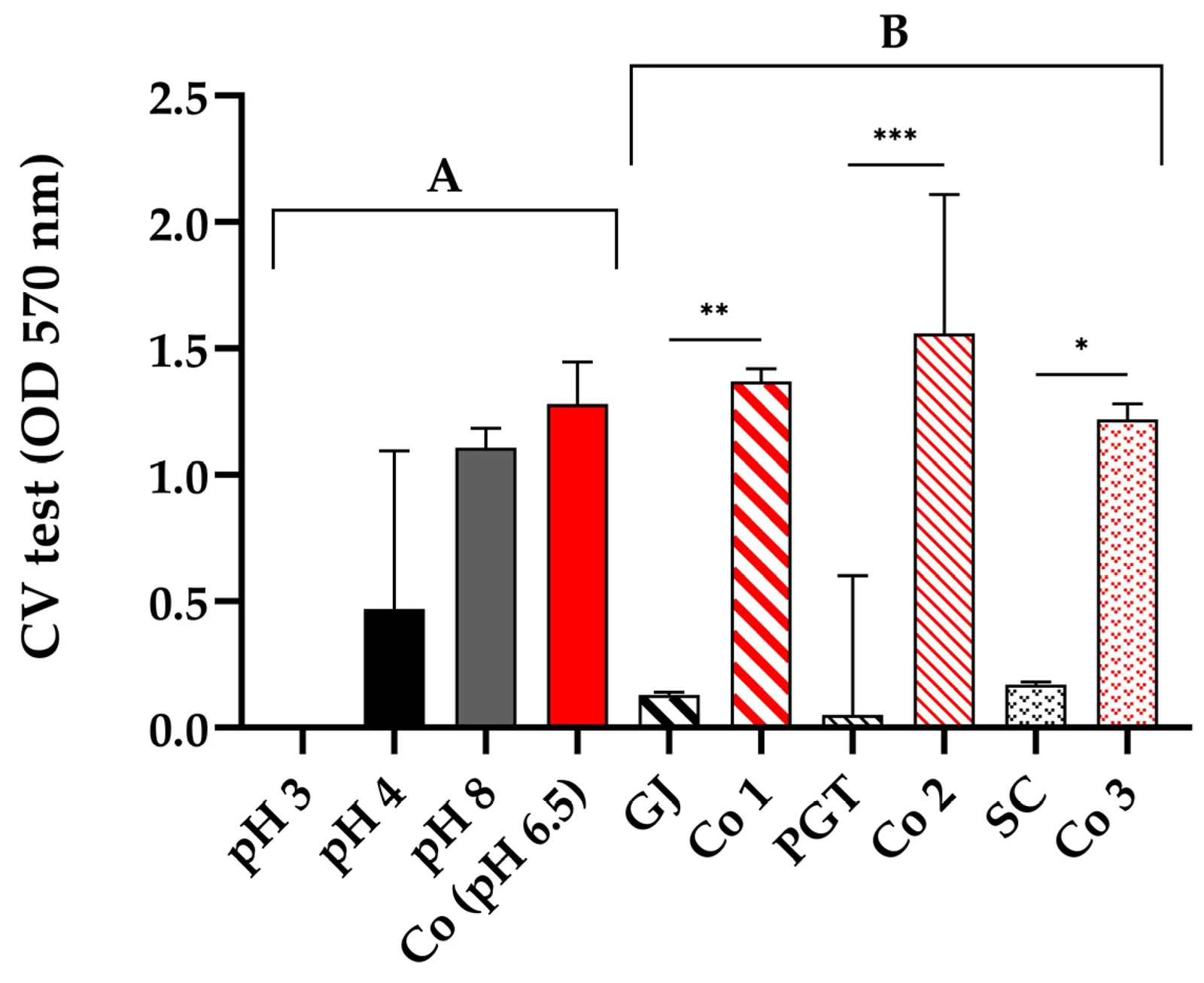
Crystal violet (CV) assay for in vitro assessment of *L. plantarum* L3 biofilms formed: (variant A) in MRS broth with different pH values, after 24 h cultivation at 37 °C and (variant B) by the L3 strain after passage through different steps of in vitro model simulated conditions of the gastrointestinal tract (GIT). * *p* < 0.05; ** *p* < 0.01 and *** *p* < 0.001. Legend: Co (pH 6.5)—Control of *L. plantarum* L3 biofilm formed in optimal growth conditions—MRS broth (pH 6.5, HiMedia, Mumbai, India); Co1, Co2 and Co3 represent the control L3 cultures corresponding to the gastric (GJ), postgastric (PGT) and simulated colon (SC) incubation stages, respectively.

Biofilm formation is considered one of the key functional traits of lactobacilli with probiotic potential, as it may facilitate adhesion, persistence, and competitive colonization within the gastrointestinal tract. Therefore, the ability of *L. plantarum* L3 to form biofilms was evaluated in vitro using a 96-well microtiter plate assay and quantified by the crystal violet (CV) staining method. The results obtained after 24 h of cultivation in MRS broth at different pH values are presented in Figure 7 (variant A). Biofilm formation was strongly influenced by the environmental pH conditions selected to mimic those encountered during gastrointestinal transit. There was no biofilm formation at pH 3.0. Increasing the pH to 4.0 enhanced biofilm formation, although biomass remained lower than at pH 8.0 and under the control conditions (pH 6.5). The highest biofilm biomass was recorded at pH 6.5, while biofilm formation at pH 8.0 was only slightly lower, indicating that strain L3 maintains a high capacity for surface attachment under neutral and mildly alkaline conditions.

The effect of sequential passage through the gastrointestinal transit model on the biofilm-forming capacity of *L. plantarum* L3 is presented in Figure 7 (variant B). To ensure an appropriate comparison, an aliquot of the same overnight culture was processed in parallel as the control. While the test samples were subjected to the successive stages of the simulated GIT model, the corresponding control cultures (Co1, Co2, and Co3) were incubated for the same period under non-stress conditions.

#### 2.2.6. Antibiotic Susceptibility

Assessment of antibiotic susceptibility is an essential criterion in the safety evaluation of lactic acid bacteria intended for probiotic, food, or biotechnological applications. The antibiotic susceptibility profile of strain L3 was evaluated using a panel of clinically relevant antibiotics according to EFSA breakpoints (Table 4).

The strain was susceptible to most of the antibiotics, including chloramphenicol, tetracycline, streptomycin, erythromycin, clindamycin, gentamicin, and kanamycin, indicating MIC values below the corresponding EFSA cut-off values. Resistance to vancomycin was observed, as growth was not inhibited at the tested concentration.

### 2.3. Whole-Genome Sequencing and Analyses of L. plantarum Strain L3

Whole-genome sequencing (WGS) of strain L3 generated a draft genome assembly comprising 119 contigs, with 98 chromosomal-associated contigs. The assembled genome had a total size of 3,260,383 bp and an overall GC content of 44.5%. A total of 3109 coding DNA sequences (CDSs) were predicted, including 64 tRNAs and 2 rRNA genes. Among the predicted CDSs, 1237 of the coding sequences represented unique genes, and 247 genes were duplicated between 2 and 11 times. Genome annotation using PROKKA identified 1330 protein sequences (42.7%) as hypothetical proteins. Assembly statistics are summarized in Table 5.

**Figure 8:**
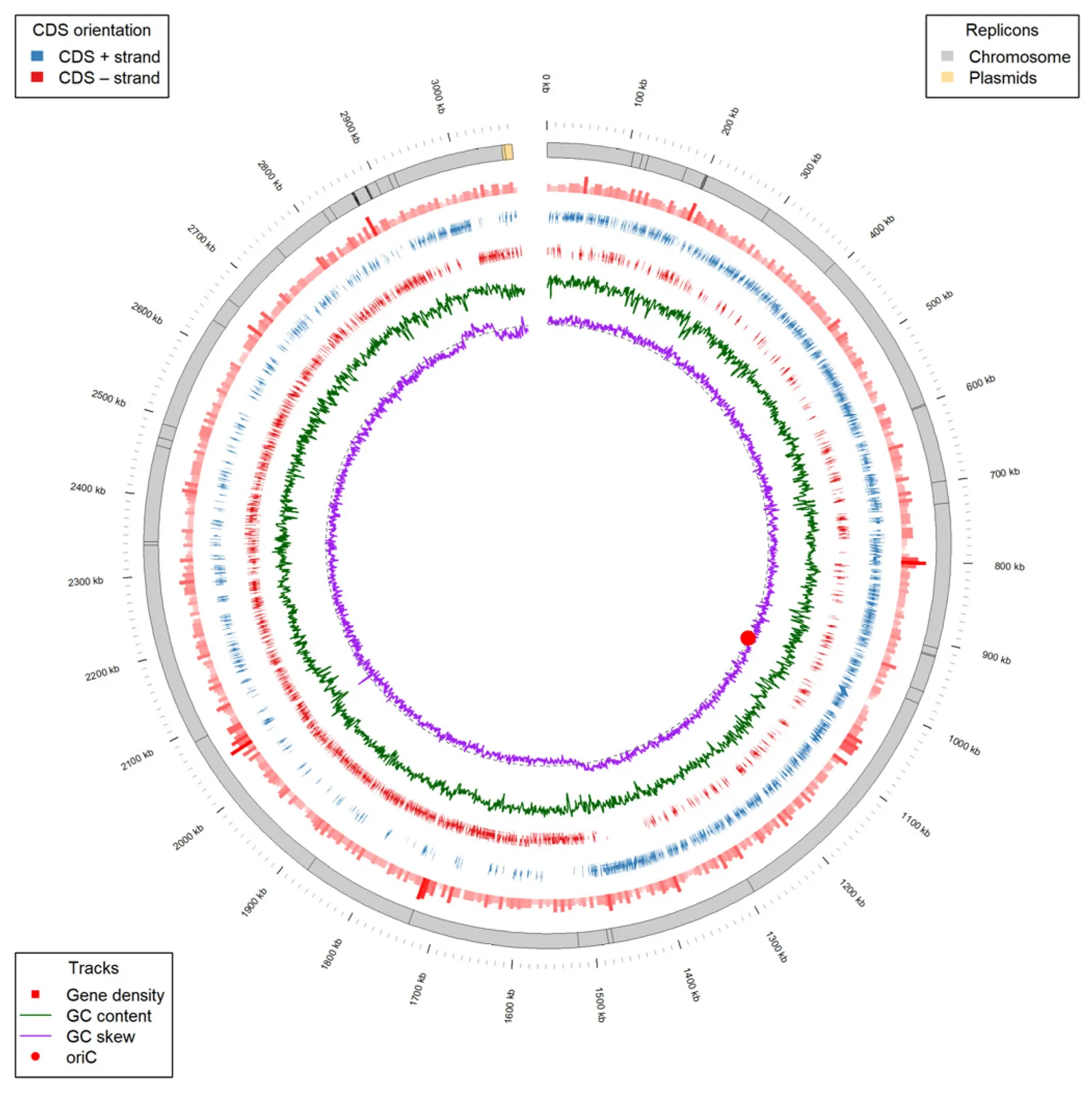
Circular genome map of the RagTag-assembled pseudo-chromosome. The genome is shown as a multi-track circular map constructed from a RagTag scaffolded pseudo-chromosome. The outermost ring displays 100 kb scale labels and 10/100 kb tick marks along the continuous genomic coordinate system. The second ring shows replicon blocks, distinguishing the chromosome (grey) from plasmids (yellow) according to their placement in the RagTag AGP file. The next ring represents gene density, calculated as the number of CDS per 5 kb window and visualized as a white to red heat gradient. Two inner rings display coding sequences (CDS) on the forward (blue) and reverse (red) strands, drawn as directional arrows and colored by COG category or strand when COG annotations were unavailable. The inner line plots show GC content (green) and GC skew (purple), computed in 5 kb windows and plotted in continuous pseudo-chromosome coordinates. The GC skew track includes a zero baseline to highlight replication-associated asymmetry. The putative origin of replication (oriC) was assigned based on the location of the *dnaA* gene and the nearest inversion point of GC skew along the pseudo-chromosome. The coordinate was converted to continuous genome coordinates using AGP-derived contig offsets and plotted as a point marker. Replicon boundary labels were added manually during figure preparation to avoid visual clutter.

A circular genome map generated for visualization purposes of the draft assembly is represented in Figure 8. Although presented in circular format, the genome assembly remained fragmented into multiple contigs and therefore should be regarded as a draft genome assembly rather than a complete closed chromosome.

Genome quality assessment indicated a high-quality draft assembly. BUSCO analysis showed 98.3% completeness based on 402 conserved single-copy orthologs, while CheckM2 estimated genome completeness at 99.5% with 0.5% contamination. The slight differences between methods reflect their distinct analytical frameworks but consistently support the high quality of the assembled genome.

#### 2.3.1. Genome Annotation and Functional Analysis

**Figure 9:**
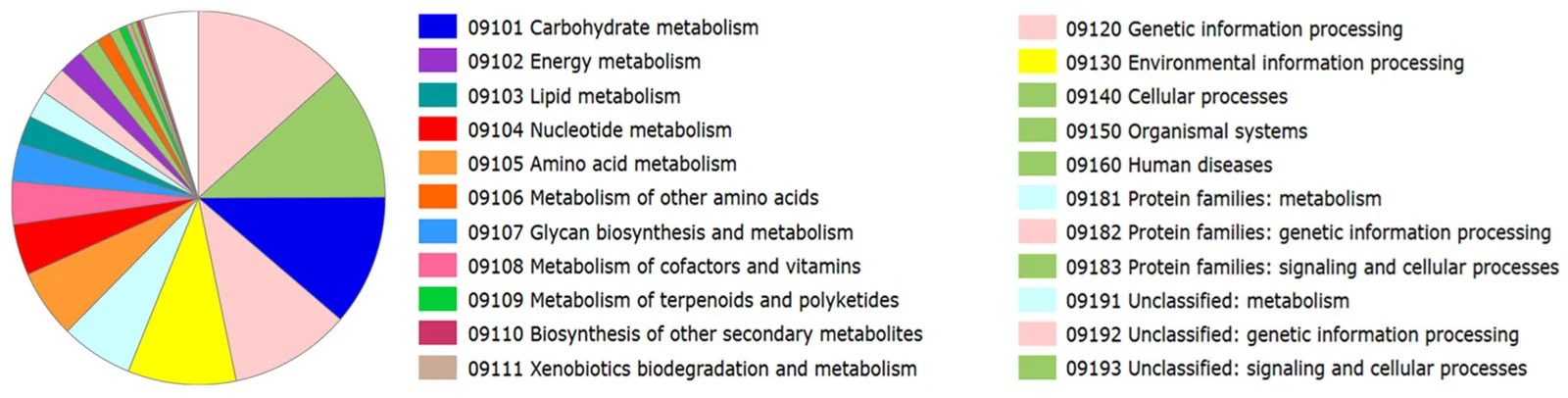
Functional annotation of genes by category.

Functional annotation of the predicted coding sequences (CDSs) was performed using the KEGG database via BlastKOALA. CDSs were assigned KEGG Orthology (KO) terms and classified into major functional categories, with 49.2% of the predicted genes assigned to KEGG categories (Figure 9).

The most abundant category was protein families associated with genetic information processing (205 genes), followed by protein families involved in signaling and cellular processes (178 genes). Genes related to carbohydrate metabolism (174 genes), genetic information processing (161 genes), and environmental information processing (144 genes) were also highly represented. Additional categories included amino acid metabolism (91 genes), nucleotide metabolism (68 genes), and cofactor and vitamin metabolism (57 genes). Furthermore, genes associated with the biosynthesis of cofactors, vitamins, prosthetic groups, and pigments were identified. The distribution of KEGG functional categories is shown in Figure 8, highlighting the functional diversity and metabolic potential of the *L. plantarum* L3 genome.

#### 2.3.2. Detection of Genes Related to Probiotic-Associated Traits

Genome analysis revealed the presence of multiple genes associated with stress tolerance, including acid and bile resistance, oxidative stress response, etc., suggesting genomic features that may contribute to adaptation and survival under gastrointestinal and fermentation-related stress conditions (Table 6).

A total of 82 genes were associated with acid and bile tolerance. Those included *dnaK*, *dnaJ*, *grpE*, and *groS*, which encode molecular chaperones that protect proteins under stress conditions. Genes encoding ATP synthase subunits and Na^+^/H^+^ antiporters (*nhaC1*, *nhaC2*) were also identified. In addition, genes encoding ATP-dependent proteases (e.g., *clp* genes) were detected. Regarding bile tolerance, the genome contained the gene encoding choloylglycine hydrolase (*cbh*), as well as genes such as *ppaC*, *cfa*, and *dps*.

Genes related to oxidative stress response included components of the thioredoxin system (*trxA*, *trxB*, *tpx*) and NADH-dependent antioxidant enzymes (*npr*). In addition, manganese transport-related genes (*mntA*, *mntB*, *mntH*) were detected. Additional information regarding the identified stress tolerance genes, including locus tags, predicted gene products, and sequence identity percentages relative to the reference genome *L. plantarum* WFC1, is provided in Supplementary Table S1.

#### 2.3.3. Detection of Bacteriocin Genes

**Figure 10:**
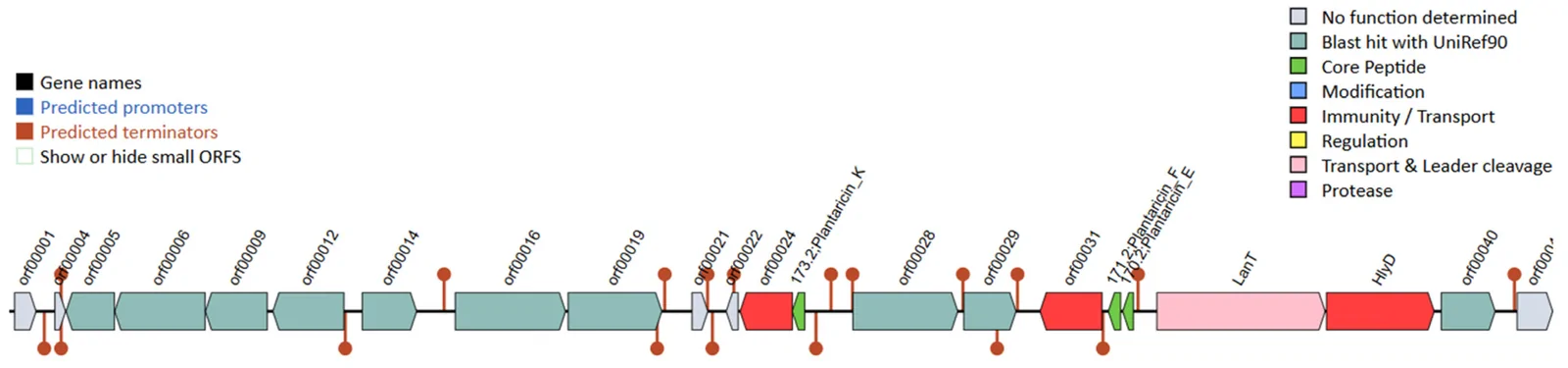
Genetic organization of the predicted plantaricin-associated gene cluster in the draft genome of *Lactiplantibacillus plantarum* L3 using BAGEL4. The promoter and terminator annotations are shown as vertical lines, as indicated in the left legend.

The genome of *L. plantarum* L3 was screened for bacteriocin-associated genes using the BAGEL4 web server. Analysis identified a plantaricin-related gene cluster containing the structural genes *plnE* and *plnF* (Figure 10).

Additionally, a gene corresponding to *plnK* was detected. However, its complementary partner, *plnJ*, which together with it forms the two-peptide plantaricin JK system, was not identified. Genes associated with bacteriocin immunity (*plnI*), transport (ABC transporter PlnG/LanT and accessory protein PlnH/HlyD), and regulation (PlnD and associated histidine kinase) were also detected within the plantaricin locus (Figure 10).

#### 2.3.4. Detection of Putative Antimicrobial Resistance Genes

To assess the presence of potential antimicrobial resistance determinants, the genome of *L. plantarum* was analyzed using the CARD database and RGI tool. No high-confidence acquired antimicrobial resistance genes were detected in the genome. All CARD/RGI and virulence-associated genes were identified on chromosomal contigs and were not associated with plasmid-derived sequences.

Two low-confidence hits were detected (*vanY* and *qacJ*) in the analyzed genome (Table 7). Both of them were identified using the protein homolog model, indicating similarity to known resistance determinants in the CARD database.

The predicted *vanY* homolog was associated with resistance to glycopeptide antibiotics, particularly vancomycin. The detected sequence showed low (31.93%) identity to the reference AMR gene. In addition, the *qacJ*-like sequence showed 40.2% identity and was associated with resistance to disinfectants and antiseptics. No other genetic determinants associated with acquired antimicrobial resistance were detected in the genome.

## 3. Discussion

### 3.1. Polyphasic Identification and In Vitro Functional Characterization of Lactiplantibacillus plantarum L3

Classical microbiological tests allow isolation of several strains from a homemade sample of a Bulgarian milk product—katak. Initial phenotyping determined them as Gram (+) lactobacilli. In several studies, classical microbiological tests were used as a step of primary isolation and putative characterization as belonging to the LAB. Based on microscopic observation, Gram (+) staining, a catalase (−) reaction, rod-shaped morphology, non-motility, non-spore-forming characteristics, and anaerobic growth at 37 °C, it was identified as a mesophilic *Lactobacillus* species. The taxonomy of the former genus *Lactobacillus* has undergone major taxonomic revision following WGS-based analyses, resulting in the establishment of 23 novel genera [15]. Our results from the application of molecular identification methods confirm the necessity of WGS for accurate LAB species identification. The combination of classical phenotypic and molecular-genetic tests, full-length 16S rRNA sequencing, and multiplex PCR, according to Torriani et al. [16], allowed the successful characterization of several isolates from katak [6,10] and white-brined cheese [5] as *L. plantarum*. The full-length 16S rRNA gene sequence classified the L3 strain in close relation with three species from the genus *Lactiplantibacillus*—*L. paraplantarum*, *L. plantarum*, and *L. pentosus*. These three species share a genetic similarity of 99.7–99.9% at the 16S rRNA gene [17] and are virtually indistinguishable using only sequence analysis of this part of the ribosomal operon. Because the 16S rRNA gene has limited discriminatory power among these closely related species, the sequence was used as supportive evidence for taxonomic assignment rather than as the sole criterion for species identification (Supplementary Table S1). Additionally, species-specific PCR using *recA* gene-targeting primers failed to produce an expected PCR product from strain L3. Therefore, whole-genome sequencing was performed, which identified the isolate as *Lactiplantibacillus plantarum*.

Whole-genome sequencing (WGS) has become the gold standard for the identification and characterization of newly isolated bacterial strains, particularly those originating from poorly studied ecological niches. Recent taxonomic revision of the former genus *Lactobacillus* has further emphasized the importance of genome-based approaches for accurate discrimination of closely related species, including members of the *Lactiplantibacillus plantarum* group. In addition to taxonomic identification, WGS enables strain traceability, comparison with previously described probiotic strains, and comprehensive safety assessment by detecting antimicrobial resistance genes, virulence-associated determinants, and other genomic features relevant to regulatory evaluation. Consequently, WGS is now widely recognized as an essential component of probiotic characterization and safety assessment, including Qualified Presumption of Safety (QPS) evaluations conducted by the European Food Safety Authority (EFSA).

Whole-genome sequencing (WGS) provides valuable insights into the taxonomic identity, functional potential, stress adaptation mechanisms, metabolic versatility, and safety-related characteristics of each newly isolated bacterial strain [18]. The genomic characteristics of strain L3 are consistent with those reported for other *L. plantarum* strains, which typically possess genomes of approximately 3.2–3.3 Mb in size, with a GC content of 44–45%. The relatively large genome size reflects the genomic complexity commonly observed in *L. plantarum*. The first fully sequenced strain, *L. plantarum* WCFS1, isolated from human saliva, possesses one of the largest genomes among lactic acid bacteria and contains numerous genes involved in carbohydrate utilization, fermentation pathways, and environmental adaptation. The strains *L. plantarum* JDM1 and ST-III have also been fully sequenced, while strains such as ATCC 14917T and KCA-1 have been partially studied [19].

Importantly, the ANI analyses provided robust species-level confirmation that strain L3 belongs to *L. plantarum*. This finding is particularly relevant because members of the *L. plantarum* group, including *L. paraplantarum* and *L. pentosus*, cannot always be reliably differentiated based solely on 16S rRNA gene sequence analysis. Therefore, genome-based approaches such as ANI offer a more accurate and reliable method for species identification and taxonomic assignment (Table 1).

As a result of their metabolism, LAB may produce various inhibitory metabolites, including organic acids, such as lactic and acetic acids, hydrogen peroxide, reuterin, diacetyl, fatty acids, bacteriocins, and bacteriocin-like substances [20], which inhibit the growth of certain Gram-positive and Gram-negative bacteria and exert specific inhibitory effects on the surrounding microflora [21,22]. As a facultative heterofermentative species, *L. plantarum* produces lactic acid together with ethanol or acetic acid, resulting in acidification of the environment and inhibition of sensitive microorganisms [23]. Our results confirmed the production of several polar metabolites by strain L3 during growth in laboratory medium MRS (Table 3).

Undesirable microbiota, including filamentous fungi, exert a significant adverse impact on the food industry, resulting in substantial losses of food products. Consequently, preservatives are widely employed to mitigate microbial contamination and extend product shelf life. In this context, the use of food-grade antagonistic microorganisms has emerged as an increasingly important and promising alternative to synthetic additives. Lactic acid bacteria exhibiting antifungal activity have strain-specific potential to inhibit spoilage-causing microorganisms, and their application as biocontrol agents has been steadily expanding globally. Variants of the agar-diffusion assay were used to assess the antifungal potential of *L. plantarum* L3. In vitro tests (Figure 2, Figure 3 and Figure 4) demonstrated that both viable cell cultures and cell-free supernatants (CFS) of strain L3 exhibited strong antifungal activity against diverse fungal taxa, including filamentous molds (*Aspergillus fumigatus*, *Fusarium oxysporum*, and *Botrytis cinerea*) as well as the yeast *Candida albicans*.

The L3 cell cultures exhibited exceptionally high antifungal activity, achieving 100% inhibition of *A. fumigatus* growth and 76% inhibition of *F. oxysporum* compared to Nystatin (antifungal control). These findings are consistent with recent studies identifying *L. plantarum* as one of the most effective lactic acid bacteria for inhibiting fungal growth in food and agricultural systems [24,25]. Previous research suggests that this antifungal effect is associated with the broad range of metabolites synthesized by this facultatively heterofermentative species, including organic acids, peptides, and other bioactive compounds [26]. This hypothesis is further supported by the results obtained against *Candida albicans*, where no significant differences were observed between the antibacterial activity of the live cultures and their corresponding cell-free supernatants. These findings indicate that the antifungal effect is primarily mediated by secreted metabolites rather than by the bacterial cells themselves. Among these metabolites, phenyllactic acid has attracted considerable attention because of its broad-spectrum activity against both filamentous fungi and yeasts. In our study, metabolomic analyses revealed increased production of phenyllactic acid (Table 3), compared with the control MRS broth [27]. The moderate yet consistent inhibition of *Botrytis cinerea* (IAA 23–55%) is in agreement with previous studies demonstrating the ability of *L. plantarum* strains to suppress the development of *B. cinerea* on fresh produce, thereby representing a promising environmentally friendly alternative to chemical fungicides [28,29].

Overall, the obtained results indicate that *L. plantarum* L3 is a promising producer of antifungal metabolites, with potential for further characterization before application in food biopreservation, bioformulation, and control of fungal contaminants. Further studies focusing on the metabolomic characterization of the cell-free supernatant and the identification of the bioactive antifungal compounds responsible for this activity are warranted.

#### Metabolomic Analysis of CFS from Exponential L3 Cultures in MRS Broth

These promising results have sparked interest in identifying the nature of the active metabolites with antagonistic effects observed in model systems. Their antifungal effect is mainly due to the production of organic acids (lactic, acetic, and phenylacetic acids), antimicrobial peptides, cyclic dipeptides, and volatile organic compounds (VOCs), which inhibit mycelial growth and sporulation [24].

Butyrate and lactic acid play an essential role in the antagonistic activity of lactobacilli through several complementary mechanisms directed against pathogenic and spoilage microorganisms. Lactic acid is the principal metabolite produced by most lactic acid bacteria. It lowers the pH of the environment, thereby creating unfavorable conditions for the growth of sensitive Gram-positive and Gram-negative bacteria. In addition to the acidification effect, the undissociated form of lactic acid can penetrate the cell membrane of pathogens. Once inside the cytoplasm, where the pH is higher, the acid dissociates and releases protons and anions, leading to intracellular acidification, disruption of the proton gradient, inhibition of enzymatic activity, and impairment of cellular energy metabolism. This mechanism is particularly important in the inhibition of pathogens such as *Escherichia coli*, *Salmonella enterica*, and *Staphylococcus aureus*. Butyrate (butyric acid), although not a major metabolite of *L. plantarum* L3, like most lactobacilli, may be produced within microbial consortia through cross-feeding interactions. Butyrate exhibits strong antimicrobial activity by damaging the cell membrane, altering membrane permeability, and inducing intracellular oxidative stress. In addition to its direct antimicrobial effect, butyrate has important physiological functions in the gastrointestinal environment, serving as a major energy source for colonocytes, supporting intestinal barrier integrity, and modulating the immune response. By stimulating mucin production and antimicrobial peptide synthesis, butyrate indirectly enhances colonization resistance against pathogens. Thus, both metabolites contribute to the probiotic potential of *L. plantarum* L3. The combined action of lactic acid, butyrate, and other organic acids, such as acetic and propionic acids, is considered a key factor underlying the antagonistic potential of lactobacilli. These metabolites act synergistically with other antimicrobial compounds, including bacteriocins, hydrogen peroxide, and diacetyl, thereby explaining the broad spectrum of inhibitory activity observed in many probiotic and food-associated strains of *Lactobacillus*/*Lactiplantibacillus*. In addition, *L. plantarum* strains may produce compounds such as diacetyl, hydrogen peroxide, and bacteriocins that further contribute to the antimicrobial effect [30,31]. The broad-spectrum activity observed in the present study may therefore result from the synergistic action of organic acids, low-molecular-weight metabolites, and bacteriocin-like inhibitory substances (BLIS). These properties identify the strain as a promising candidate for further investigation of its antimicrobial metabolites and their potential applications in food biopreservation. The activity of other katak isolates was determined toward the same range of pathogens [6]. Published results indicated that activity against *S. aureus*, *S. Typhimurium*, *L. monocytogenes*, and *P. aeruginosa* was probably due to the production of Pln F bacteriocin [18]. The activity of other katak isolates was determined toward the same Various factors, including nitrogen oxides, herbicides, xenobiotics, ozonation, radiation, and certain metals, can induce oxidative stress in the intestinal microbiota [32]. Therefore, the antioxidant activity of L3 was evaluated in vitro using live exponential-phase cells (24 h culture) and cell-free filtered supernatants (CFS). Radical scavenging activity was determined against DPPH and ABTS radicals [33].

The beneficial properties of postbiotics/metabiotics (CFSs) produced during *Lactobacillus* fermentation have been highly discussed [34,35]. These may act as natural antioxidants, able to support the host defense system against oxidative stress (OS) [36]. Similar activity has been observed for exopolysaccharides [37], cell wall fragments (CWF) [37,38], short-chain fatty acids [23], and organic acids [39].

The present study confirmed a promising antioxidant potential of *L. plantarum* L3 and supports previous reports describing the antioxidant activity of LAB-derived supernatants [40]. Considerable differences in antioxidant activity among LAB strains have been reported previously. Chooruk et al. (2017) demonstrated DPPH radical-scavenging activity ranging from 11.3% to 53.2% in cell lysates and intact cells, with approximately 23% activity reported for *L. plantarum* strains [41].

Our results correlate with other studies in this field. Spent cultures of *L. plantarum* strain MA2 isolated from Tibetan kefir (85.57 ± 0.44%) and whole cells (83.93 ± 1.54%) showed very high DPPH-scavenging activity [42].

*L. plantarum* L3 is a producer of high-level lactic acid and SCFA acids during the 24/48 h laboratory fermentations in MRS broth (HiMedia, Mumbai, India). The summary of GS-MS chromatographic analyses is presented in Table 3. Although the main product of *L. plantarum* is lactic acid, its metabolic flexibility allows it to participate in complex fermentation pathways leading to the production of various polar metabolites and organic acids depending on the available sugars and environmental conditions.

The results indicate that both low-molecular-weight metabolites and exopolysaccharide components contribute significantly to the overall antioxidant capacity. The antioxidant activity of EPS is consistent with previous findings on lactic acid bacteria [43]. In contrast, the protein extract exhibited comparatively lower antioxidant activity, suggesting that not all proteinaceous components are equally effective at scavenging radicals. The results suggest that high-molecular-weight components play a major role in the antioxidant properties of this strain. In a study by Sun et al. (2025), supernatants exhibited higher antioxidant activity than whole cells as evaluated by the DPPH method (90.95% vs. 30.57%) [44]. Our results from the ABTS radical-scavenging assay also showed lower antioxidant activity in whole cells (29%), consistent with previously reported findings [44].

Survival during gastrointestinal transit is considered a key functional characteristic of candidate probiotic strains. Upon entering the gastrointestinal tract (GIT), bacteria are exposed to multiple stress factors, including low pH, digestive enzymes, and bile salts, which may substantially reduce cell viability. Therefore, the ability to withstand these conditions is commonly regarded as an important prerequisite for probiotic functionality. Biofilm formation is an important probiotic trait, as *Lactobacillus* biofilms contribute to intestinal epithelial barrier integrity, support mucosa-associated immune functions, and help prevent pathogen colonization [45,46].

In the present study, *L. plantarum* L3 demonstrated high tolerance to simulated gastrointestinal conditions. The strain exhibited a moderate reduction in viability during the gastric phase (approximately 0.81 log CFU/mL), while no additional loss of viability was observed during the subsequent modified intestinal phase. During the simulated colon phase, only a slight reduction (0.18 log CFU/mL) was detected. Overall, the strain maintained high viable cell counts throughout the entire in vitro gastrointestinal transit (Figure 6).

In the present study, biofilm formation by *L. plantarum* L3 was strongly influenced by environmental pH (Figure 7 variant A). Exposure to simulated gastric juice (GJ) and the post-gastric transition stage (PGT) resulted in a pronounced reduction in biofilm formation compared with their respective controls (Figure 7 variant B), indicating that the model gastrointestinal stress conditions significantly impaired the biofilm capacity of strain L3. In contrast, the control cultures maintained high biofilm-forming ability throughout the experiment. The sequential stresses incorporated into the GIT model inhibit biofilm development and confirm that biofilm formation is highly responsive to changes in the gastrointestinal environment. Sequential exposure to the simulated gastrointestinal tract significantly reduced biofilm formation compared with the corresponding controls, indicating that gastrointestinal stresses impair the biofilm formation of strain L3 (Figure 7 variant B). Biofilm formation is known to be strain- and species-specific. This response may also reflect the combined effects of acid, digestive enzymes, and bile salts, the latter of which have been shown to reduce exopolysaccharide synthesis and consequently biofilm formation [47].

Low gastric pH is considered one of the major obstacles for probiotic bacteria [48]. Gastric pH may vary between 1.3 and 4.5 depending on fasting or postprandial conditions [43,44]. Fernández de Palencia et al. (2008) reported survival rates above 80% for *Lacticaseibacillus paracasei* and *Lactobacillus acidophilus* incubated at pH 3.0 [49]. Similarly, *L. plantarum* is known to be well adapted to gastrointestinal conditions, which explains its frequent occurrence in the human digestive tract [50].

Margalho et al. (2021) evaluated 220 isolates from Brazilian artisanal cheeses for tolerance to low pH and bile salts and found that 41.8% were resistant to acidic conditions (pH 2.0–3.0) [51]. Most resistant isolates belonged to *L. plantarum* (46.7%), followed by *L. brevis*, *L. paracasei*, *Lactococcus lactis*, *Lacticaseibacillus rhamnosus*, etc. The ability of these strains to tolerate acidic conditions may be related to their source of isolation, since fermented foods naturally reach low pH values due to LAB metabolic activity [51]. For example, artisanal Brazilian cheeses may reach pH values around 4.7, creating selective pressure favoring acid-resistant bacteria [52].

These findings support the observed tolerance of L3 to gastrointestinal stress conditions and further confirm its potential as a candidate probiotic.

Although many members of lactobacilli are generally recognized as safe, screening for antibiotic resistance remains necessary to exclude the presence of acquired and potentially transferable resistance determinants that could contribute to the spread of antimicrobial resistance. The antibiotic susceptibility profile of strain L3 supports its safety for potential probiotic and food applications.

The strain was susceptible to all tested antibiotics except vancomycin.

Susceptibility to chloramphenicol, tetracycline, erythromycin, clindamycin, gentamicin, streptomycin, and kanamycin is particularly important, as these antibiotics represent clinically relevant classes often associated with acquired and mobile resistance determinants in lactic acid bacteria. The low MIC values observed for strain L3 indicate the absence of acquired resistance mechanisms to these compounds.

In contrast, resistance to vancomycin is a well-known intrinsic characteristic of *Lactobacillus* species and related lactic acid bacteria. This intrinsic resistance is associated with the natural structure of the peptidoglycan precursors, which reduces the binding affinity of glycopeptide antibiotics. Importantly, this type of resistance is chromosomally encoded, non-transferable, and not considered a safety concern under EFSA guidelines.

The antibiotic susceptibility profile of *L. plantarum* L3 shows sensitivity to most tested antibiotics, including chloramphenicol, tetracycline, streptomycin, and erythromycin. The observed resistance to vancomycin is consistent with the intrinsic resistance typical of *Lactobacillus* strains; the susceptibility pattern supports the potential safe use of this strain.

### 3.2. Whole-Genome Analysis and Identification of Putative Functional and Safety-Related Genetic Determinants of L. plantarum L3

The KEGG annotation profile of *L. plantarum* L3 (Figure 9) reveals a metabolically diverse genome, with nearly half of the predicted genes assigned to known functional pathways. The majority of genes are associated with genetic information processing and signaling-related protein families. The categories “Protein families: genetic information processing” (205 genes) and “Genetic information processing” (161 genes) represent different annotation classifications generated by BlastKOALA. The first summarizes genes according to protein families, whereas the latter summarizes genes according to functional pathways; therefore, different numbers of annotated genes are expected. The high representation of carbohydrate metabolism genes is consistent with the fermentative nature of *L. plantarum* and indicates the strain’s ability to utilize a wide range of carbon sources [53]. This metabolic flexibility is particularly important for survival in nutrient-variable environments such as fermented foods and the gastrointestinal tract.

The presence of genes involved in environmental information processing, including membrane transporters and signal transduction systems, further suggests the capacity to sense and respond to external stimuli [44]. Such capabilities are associated with enhanced adaptability [44]. Similarly, the presence of pathways related to amino acid metabolism, nucleotide metabolism, and cofactor and vitamin biosynthesis suggests a relatively broad metabolic repertoire that may support both environmental adaptation and fermentation-associated functions.

Overall, the genomic profile of strain L3 is consistent with the metabolic versatility commonly observed in *L. plantarum*. However, it should be noted that KEGG annotation is based on genomic predictions and does not provide direct evidence of gene expression or metabolic activity under specific environmental conditions. Therefore, experimental validation would be required to confirm the functionality of the predicted pathways.

The presence of multiple stress-response genes suggests that *L. plantarum* L3 possesses genetic determinants potentially associated with survival under gastrointestinal and fermentation stress conditions, biofilms and oxidative stress conditions (Supplementary Table S1).

Genes encoding molecular chaperones (*dnaK*, *dnaJ*, *grpE*, *groS*) are known to contribute to protein stability under acid stress, thereby supporting adaptation to low-pH environments. Synthesis of molecular chaperones is induced under acid stress conditions, including exposure to moderately acidic environments (e.g., pH 4.5), as previously observed for *Lactococcus lactis* [54]. Similarly, ATP synthase systems and Na^+^/H^+^ antiporters (*nhaC_1*, *nhaC_2*) are commonly involved in intracellular pH regulation, which is essential for bacterial survival in acidic conditions (Table 6) [55].

Bile tolerance is supported by the presence of a gene encoding choloylglycine hydrolase (*cbh*), which plays a key role in bile salt deconjugation. Additional bile-associated genes such as *ppaC*, *cfa*, and *dps* were also identified. The *ppaC* gene has been associated with cellular stress adaptation and maintenance of physiological functions under adverse environmental conditions, whereas *cfa* is a synthase that enhances lipid synthesis. The *dps* gene encodes a DNA protection during starvation protein and is associated with enhanced protection under stress conditions. Proteins of the choloylglycine hydrolase (also called bile salt hydrolase) family (such as *cbh*) catalyze the deconjugation of bile salts, a mechanism frequently associated with bile tolerance in lactic acid bacteria [56].

Strains with high resistance to oxidative stress are able to survive under harsh conditions and exhibit strong antioxidant activity. Several genes involved in oxidative stress response were identified in strain L3, including the thioredoxin system genes (*trxA*, *trxB*, and *tpx*) and the gene encoding NADH peroxidase (*npr*). The genes exhibited very high nucleotide sequence identity to the corresponding genes of the probiotic reference strain *Lactiplantibacillus plantarum* WCF1 (Supplementary Table S1) These genes constitute major components of the antioxidant defense machinery in lactobacilli by maintaining intracellular redox homeostasis and scavenging reactive oxygen species (ROS), thereby contributing to enhanced tolerance to oxidative stress [57].

In agreement with findings reported for *Lacticaseibacillus paracasei* SP5 [55], the presence of manganese transport-related genes (*mntB* and *mntH*) may contribute to oxidative stress resistance. Intracellular accumulation of Mn(II), mediated by manganese transport systems, has been suggested to functionally compensate for the absence of superoxide dismutase activity in certain lactic acid bacteria.

Whole-genome sequencing of the investigated *L. plantarum* strain L3 revealed the presence of the *luxS*, *comC*, *comEA* and *comEC* genes, showing a high degree of sequence similarity to their homologues in the reference strain *L. plantarum* WCF1 (Table S1). The identification of these genes provides genomic evidence supporting the probiotic-associated phenotype observed in vitro. Although the presence of these genes alone does not prove their expression or functionality, their conservation in a well-characterized *L. plantarum* genome suggests that they may contribute to mechanisms associated with bacterial persistence and adaptation. The *luxS* gene encodes S-ribosylhomocysteine lyase, an enzyme involved in the activated methyl cycle and in the production of autoinducer-2 (AI-2), a universal quorum-sensing signaling molecule. In lactobacilli, the LuxS/AI-2 system has been associated with the regulation of biofilm formation, extracellular polysaccharide production, adhesion to epithelial surfaces, and tolerance to environmental stresses, including low pH and bile salts [58]. These properties are directly related to successful gastrointestinal colonization and are regarded as important determinants of probiotic functionality [59].

Bacteriocins are an important class of antimicrobial peptides, produced by LAB, and contribute to barrier mechanisms against other bacteria, including pathogens. Genome mining of strain L3 identified a plantaricin-associated gene cluster containing structural, regulatory, transport, and immunity-related genes, suggesting a genetic capacity for bacteriocin synthesis (Figure 10). The identification of a plantaricin-associated locus in *L. plantarum* L3 is consistent with previous reports describing the widespread occurrence of bacteriocin systems in this species [44].

Plantaricin EF is a class IIb (non-lantibiotic) bacteriocin that requires the synergistic action of both peptides for optimal antimicrobial activity, typically targeting closely related Gram-positive bacteria. The presence of *plnE* and *plnF* is consistent with previous reports describing plantaricin EF systems in food-associated *L. plantarum* isolates. Three *L. plantarum* strains, isolated from food, have been reported to encode plantaricin systems, including *plnEF* and *plnJK* gene pairs [60].

In contrast, only *plnK* was detected within the plantaricin JK system, whereas the complementary *plnJ* gene was not identified. Since plantaricin JK activity depends on the combined action of both peptides, the absence of *plnJ* may limit or abolish the functionality of this system, as previously reported [61].

The identification of immunity (*plnI*), transport (ABC transporter PlnG/LanT and accessory protein PlnH), and regulatory genes (*plnD* and *plnS*) further supports the presence of a conserved plantaricin-associated locus. Immunity genes likely provide self-protection against the produced bacteriocin, while transport genes are required for secretion of bacteriocin peptides and regulation in response to environmental signals.

However, it is important to note that these findings are based on in silico predictions. The presence of bacteriocin-related genes does not necessarily confirm their expression or the production of active antimicrobial peptides. Therefore, further experimental validation, including gene expression analysis, is required to confirm the functional capacity of this system.

The CARD/RGI analysis was performed to assess the presence of antimicrobial resistance determinants and to evaluate the safety profile of *L. plantarum* L3 (Table 7).

No acquired antimicrobial resistance genes were detected in the genome. Although two putative homologs, *vanY* and *qacJ*, were identified, both exhibited relatively low sequence identity to known resistance determinants (31.93% and 40.2%, respectively) (Table 7).

The detection of a *vanY*-like sequence should be interpreted with caution. Its low sequence identity suggests that it is more likely to represent a distant homolog than a functionally acquired antimicrobial resistance determinant. While *Lactiplantibacillus* species are generally considered intrinsically resistant to vancomycin, this intrinsic phenotype is independent of the detection of a low-similarity *vanY*-like sequence. It should not be interpreted as evidence supporting its functionality.

Similarly, the *qacJ*-like sequence may be associated with tolerance to disinfectants and environmental stress conditions. However, its functional significance remains uncertain due to the relatively low sequence identity to characterized resistance determinants and the absence of supporting genomic evidence.

No acquired determinants were detected. Nevertheless, genomic predictions should be interpreted alongside phenotypic antimicrobial susceptibility testing to provide a comprehensive assessment of strain safety.

## 4. Materials and Methods

### 4.1. Isolation and Polyphasic Taxonomy Identification of L3 Strain from Katak

A homemade katak sample obtained from a small farm near Lukovit, Bulgaria (43°12′ N, 24°10′ E), was used to isolate lactic acid bacteria (LAB). Ten grams of the sample were homogenized in sterile physiological saline (0.85% *w*/*v* NaCl), serially diluted, and plated onto Rogosa agar (HiMedia, Mumbai, India) using the Koch plating method. The plates were incubated at 37 °C for 48 h under microaerophilic/anaerobic conditions. The isolates were subsequently purified and cultured twice in De Man Rogosa Sharp (MRS) broth (pH 6.5) (HiMedia, Mumbai, India) under anaerobic conditions at 37 °C. Presumptive LABs were pre-selected based on colony and morphology, Gram (+) staining, catalase and oxidase negative test results, microscopic observation, and negative results for spore formation. For subsequent testing, the isolates were stored at −20 °C in MRS broth enriched with 20% (*v*/*v*) sterile glycerol (SoPharmacy, Sofia, Bulgaria), with a 4–6-h pre-cooling/acclimatization phase. In addition, duplicates of the stocks were prepared in cryotubes from a commercially available sterile storage system. In addition, duplicate stocks in cryovials from a commercial sterile system for conservation of microbiological strains “Cryoinstant: Porous beads for microbiological culture preservation” (Deltalab, Barcelona, Spain) were made for long-term storage at −80 °C.

DNA from strain L3 was extracted using the HiPurA™ Genomic DNA Purification Kit (HiMedia, Mumbai, India) according to the manufacturer’s instructions. The full-length 16S rRNA gene was amplified using Illustra™ PuReTaq™ Ready-To-Go™ PCR Beads (Amersham Biosciences, Amersham, UK) according to the manufacturer’s recommendations. Universal bacterial primers 27F and 1492R (Macrogen Europe, Amsterdam, The Netherlands) were used for PCR amplification. The amplified PCR product was sequenced by Macrogen Europe (a branch office of South Korea-based Macrogen Inc., Seoul, Republic of Korea).

The obtained sequences were manually processed using Sequence Scanner Software v1.0 (Applied Biosystems, Foster City, CA, USA) to remove PCR primer-binding sites. The edited sequences were then compared with nucleotide sequences available in the NCBI GenBank database using the Basic Local Alignment Search Tool (BLAST).

Species-level identification within the *Lactiplantibacillus plantarum* group was further confirmed by multiplex PCR targeting the *recA* gene, following the protocol described by Torriani et al. (2001) [16]. Genomic DNA extracted from the reference strains *Lactiplantibacillus paraplantarum* ATCC 700211^T^, *Lactiplantibacillus plantarum* ATCC 14917^T^ and *Lactiplantibacillus pentosus* ATCC 8041^T^ was used as a positive control in the multiplex PCR assays.

### 4.2. In Vitro Evaluation of Functional Characteristics and Probiotic Potential of Strain L3 Isolated from Katak

#### 4.2.1. In Vitro Antibacterial Activity of *L. plantarum* Strain L3

- Indicator microorganisms, media, and culture conditions

The antimicrobial activity of strain L3 was evaluated against the following indicator microorganisms: *Escherichia coli* *ATCC 25922*, *Staphylococcus aureus* CRM 06571L, *Streptococcus mutans* DSMZ 20523, *Pseudomonas aeruginosa* PAO1 WDCM VT 000263, and *Chromobacterium violaceum* DSMZ 30191. Stock cultures were maintained at −20 °C in appropriate culture media, supplemented with 20% (*v*/*v*) glycerol. Prior to the assays, the indicator strains were activated by cultivation at 37 °C for 18–24 h. *E. coli*, *S. aureus*, and *S. mutans* were cultivated in Brain Heart Infusion (BHI) broth (Difco, Detroit, MI, USA), whereas *P. aeruginosa* was cultivated in Tryptic Soy Broth (TSB; DifcoLaboratories, Detroit, MI, USA). Sabouraud Dextrose broth and Sabouraud Dextrose agar (HiMedia, Mumbai, India) were used for anti-*Candida albicans* tests.

- Preparation of cell-free supernatants from *L. plantarum* L3

The *Lactobacillus* L3 strain was cultivated in de Man, Rogosa and Sharpe (MRS) broth at 37 °C for 24 h in aNUVE EN 400-270 L incubator (Ankara, Turkey). After incubation, the bacterial culture was centrifuged at 9000 rcf for 10 min (Hermle, Wehingen, Germany) to remove bacterial cells. The resulting supernatant was carefully collected and filtered through a sterile 0.22 μm membrane filter (MS^®^ CA Syringe Filter, Membrane Solutions, Auburn, WA, USA) to obtain sterile cell-free supernatants (CFS). The obtained CFS was stored at 4 °C and used immediately for antimicrobial activity assays. CFSs were used in the evaluation of antagonistic activity and radical scavenging activity tests.

- Agar Diffusion Assay

To determine antibacterial activity, the agar-diffusion method was used, modified according to Tagg and Mac Given (1971) [62], with the following variations.

For initial screening of antimicrobial activity, acidic (aCFS) and neutralized cell supernatants (nCFS) from the L3 strain were cultured in MRS broth (24–48 h, 37 °C) in a NUVE-270 L thermostat at 37 °C under anaerobic conditions. The supernatants were collected by centrifugation (9000 rcf, Hermle, Wehingen, Germany, for 10 min), followed by filtration through a 0.22 μm bacterial siringe filters (Merck, Millipore, Germany). The neutralized nCFS were adjusted to a pH of 5.5–6.0 ± 0.2 with 5 M NaOH.

Tests with 10% (*w*/*v*) skim milk (Serva, Spain) or freshly prepared soy milk (freshly prepared in the laboratory), inoculated with a 2% (*v*/*v*) inoculum of *Lactobacillus* culture L3 (exponential culture—24 h at 37 °C on MRS), were also used in the tests. From the resulting sample, whey fractions (WF) were collected by centrifugation (5000 rpm, 10 min) (Hermle, Wehingen, Germany) and used in the agar-diffusion assay.

#### 4.2.2. Antifungal Activity of *L. plantarum* L3

Test fungal strains *Aspergillus fumigatus*, *Candida albicans*, *Botrytis cinerea*, and *Fusarium oxysporum* were obtained from the culture collection of the Mycology Department at the Stephan Angeloff Institute of Microbiology, Bulgarian Academy of Sciences (BAS). The strains were maintained on potato dextrose agar (PDA) plates (Oxoid, Hampshire, UK) at 29 °C and subcultured monthly until sporulation.

In vitro tests were conducted using a modified version of the protocol by Tropcheva et al. (2014), [13] employing two methods: the monolayer (only PDA—2% *w*/*v* agar) method (Oxoid, Hampshire, UK) and the two-layer agar method (MRS agar + PDA 2% *w*/*v*). According to the two-layer method, Petri dishes were partially half-filled with ~10 mL cooled MRS agar (HiMedia, Mumbai, India), into which an overnight culture of L3 (24 h, 37 °C) was inoculated. The plates were incubated for 24 h at 37 °C. Subsequently, a layer of PDA medium was poured into the MRS agar layer, and the corresponding test fungus was inoculated to obtain a single colony. In the single-layer method, acidic cell-free supernatant (aCFS) from the LAB strain (10% *v*/*v*) was incorporated into PDA medium. The test fungus was inoculated in the center of the Petri dish to obtain a single colony. The incubation was at room temperature (23–25 °C) for 6–11 days in the presence of 10% *v*/*v* aCFS from LAB. The diameters of the growing colonies were measured daily. The experiment was conducted in triplicate. For each treatment variant, a control containing medium supplemented with spore suspension was used. The degree of inhibition was determined based on the colony diameter and expressed as a percentage relative to the untreated control using the Index of Antifungal Activity (IAA)

IAA (%)=(1−Dsample/Dcontrol)×100

The agar diffusion method was also used for the antifungal activity assay. Cell-free supernatants obtained during the cultivation of *Lactiplantibacillus plantarum* L3 in MRS broth (HiMedia, Mumbai, India) at 37 °C were evaluated against *Candida albicans*. Anti-*Candida* activity was determined on Sabouraud agar by measuring the diameters of the inhibition zones. As a positive control, nystatin (10 mg/mL) was used. Sterile MRS broth was used as a negative control.

All the experiments were performed in triplicate.

#### 4.2.3. Metabolomic Analysis of Cell-Free Supernatants of *L. plantarum* L3

Lyophilized CFS samples (1.0 mL) were prepared for analysis of polar and non-polar metabolites using established derivatization protocols. Internal standards (ribitol and nonadecanoic acid, 1.0 mg/mL; 50 µL each) were added prior to extraction. Subsequently, 1.0 µL of the derivatized sample was injected into an Agilent 7890A gas chromatograph coupled to an Agilent 5975C mass spectrometer. Separation was performed on an HP-5MS column (length 30 m × diameter 0.32 mm × film thickness 0.25 µm) under optimized temperature programs and modes for polar and non-polar compound analysis. Compounds were identified by comparing retention times and Kovats retention indices (RI) with authentic standards, as well as by matching mass spectra against the Golm Metabolome Database and the NIST 2008 mass spectral library (National Institute of Standards and Technology, USA).

#### 4.2.4. Antioxidant Activity of *L. plantarum* Strain L3 from Katak

The antioxidant activity of strain L3 was evaluated using the DPPH and ABTS radical-scavenging assays. DPPH free radical scavenging activity was determined using 1,1-diphenyl-2-picrylhydrazyl (DPPH) solution (0.2 mM; Sigma-Aldrich, St. Louis, MO, USA), according to the method described by Brand-Williams et al. (1995) [63], with a modification to adapt the assay to the specificity of the tested samples (cells and CFS). Following incubation of the reaction mixture in the dark at room temperature, absorbance was measured at 517 nm.

The ABTS radical scavenging activity was evaluated using 2,2′-azino-bis(3-ethylbenzothiazoline-6-sulfonic acid) (ABTS) according to the method of Re et al. (1999) [64]. The absorbance of the reaction mixture after incubation in the dark at room temperature was measured at 734 nm.

Ascorbic acid (0.1 mg/mL) served as a positive control. Radical scavenging activity (%) was calculated as follows:

DPPH activity (%)=(ODControl−ODSample)/ODControl×100

ABTS activity (%)=(ODControl−ODSample)/ODControl×100

- Preparation of concentrated CFS (<3000 kDA and >3000 kDA)

Ultrafiltration of exponential cell-free supernatants was carried out to obtain two fractions—below and above 3000 Da—using Amicon R Ultra-15 (Merck, Millipore, Germany) centrifugal filters at 4 °C.

- Preparation of Exopolysaccharides

Exopolysaccharides (EPS) were obtained from the cell-free supernatant of L3 cultures using the cold ethanol precipitation method. Following centrifugation to remove bacterial cells, the supernatant was collected and mixed with four volumes of cold absolute ethanol. The mixture was incubated at 4 °C for 24 h to allow EPS precipitation. The polymers were then separated by centrifugation at 4000× *g* for 25 min at 4 °C, washed with cold ethanol, and re-suspended in distilled water before further experiments. The resulting crude EPS preparation was used for subsequent analyses.

- Preparation of protein crude extract

Protein extract was obtained from the cell-free supernatant of L3 culture, late exponential phase, by the classical ammonium sulphate precipitation method. A 50% *w*/*v* saturation was applied for 4 h at 4 °C with magnetic stirring. The pellets were harvested by centrifugation at 4 °C at 18,000 rcf for 20 min. (Hermle centrifuge, Wehingen, Germany) and washed with sterile distilled water. The suspended pellets in distilled water (1/10 from the initial volume) were dialyzed overnight at 4 °C to remove the cells. The antioxidant activity of dialyzed samples containing proteins was determined by DPPH and ABTS methods.

#### 4.2.5. Survival Under Simulated Gastrointestinal Conditions

Survival under simulated gastrointestinal conditions was evaluated using a modified sequential model based on the method described by Charteris et al. (1998) [14]. The model consisted of sequential exposure of bacterial cells to simulated gastric, post-gastric, and colon-like conditions, following the algorithm used by an automated system of dynamic GIT passage. Simulated gastric juice (GJ) was prepared by dissolving pepsin (0.1% *w*/*v*; Sigma-Aldrich, USA) in sterile sodium chloride solution (0.5% *w*/*v*), with the pH adjusted to 3.0 using 37% HCl. Following the gastric phase, cells were transferred to simulated post-gastric transition (PGT) conditions consisting of pancreatin (0.1% *w*/*v*; HiMedia, India) and bile salts (1.5% *w*/*v* oxgall; Difco, USA), sterilized by membrane filtration (Millipore, 0.45 µm), with the final pH 6.0. This modification was introduced to simulate the transitional conditions encountered immediately after gastric emptying, when acidic chyme enters the proximal small intestine and undergoes gradual neutralization by pancreatic bicarbonate secretion. Simulated colon-like (SC) conditions were established using MRS broth adjusted to pH 8.0 with 5 M NaOH and supplemented with porcine mucin (0.1% *v*/*v*; Sigma-Aldrich, USA).

Two equal aliquots (0.2 mL each) of the washed L3 bacterial cell suspension prepared in sterile physiological saline and containing >10^9^ CFU/mL were used. One aliquot was subjected to the simulated gastrointestinal model (designated as the sample), whereas the second aliquot was maintained as an untreated control. The experimental aliquot was inoculated into 1.0 mL of simulated gastric juice and incubated at 37 °C for 180 min. After completion of the gastric phase, cells were recovered by centrifugation, transferred into simulated PGT conditions, and incubated at 37 °C for 240 min. Cells recovered after the intestinal phase were subsequently exposed to colon-like conditions containing mucin and incubated for an additional 480 min. In parallel, untreated control cells were incubated at 37 °C in sterile physiological saline solution during the gastric and PGT phases and in MRS broth (pH 6.5) during the colon-like phase.

The number of viable cells at each stage of the simulated gastrointestinal model was determined using the Koch plate count method and expressed as log CFU/mL. Viability was calculated according to the following equation:

$$Viability\;(as\log CFU/mL)=logN_{1}/logN_{0}$$

where N_0_ represents the initial viable cell count prior to exposure, and N_1_ represents the viable cell count after each simulated gastrointestinal phase.

#### 4.2.6. Antibiotic Susceptibility

Biofilm formation by strain L3 under different pH conditions (3.0, 4.0, 8.0, and pH 6.5 used as the control condition), as well as following each incubation step of the sequential model for assessing gastrointestinal transit tolerance (Section 4.2.5), was evaluated using the crystal violet (CV) assay as previously described by Dobreva et al. (2023) [10]. Briefly, cells recovered from samples exposed to simulated gastrointestinal tract (GIT) conditions, together with untreated L3-as control cells, were inoculated in triplicate (10%, *v*/*v*) into the wells of sterile 96-well microtiter plates. The plates were incubated at 37 °C for 24 h (in a 5% CO_2_ atmosphere), after which the wells were washed five times with sterile physiological saline to remove planktonic cells. The attached biofilms were fixed with 100% methanol (analytical grade) for 15 min and stained with 0.1% (*v*/*v*) crystal violet solution for an additional 15 min. Subsequently, the wells were washed five times with sterile physiological saline to remove excess, unbound crystal violet. The retained dye was solubilized in 70% ethanol, and the biofilm biomass was quantified by measuring the optical density at 570 nm (OD570) using a microplate reader (INNO, Seongnam-si, Republic of Korea).

#### 4.2.7. Antibiotic Susceptibility

The antibiotic susceptibility profile of the *Lactobacillus* L3 strain was evaluated using commercial MIC gradient strips (Easy MIC™ Strip, HiMedia Laboratories, India) containing decreasing concentrations of selected antibiotics. The antibiotics tested were chloramphenicol, tetracycline, streptomycin, erythromycin, clindamycin, gentamicin, kanamycin, and vancomycin. Briefly, an overnight culture of L3 (24 h in MRS at 37 °C) was subcultured into fresh MRS broth and incubated for 4 h. The bacterial suspension was adjusted to McFarland standard 2 and uniformly inoculated onto MRS agar plates. MIC strips were aseptically placed on the agar surface, and plates were incubated at 37 °C for 24 h. The minimum inhibitory concentration (MIC) was determined according to the manufacturer’s instructions and defined as the point at which the inhibition ellipse intersected the scale on the strip.

The obtained MIC values were interpreted according to the microbiological cut-off values established by the European Food Safety Authority (EFSA) for lactic acid bacteria. Susceptibility or resistance was assigned based on comparison of the measured MIC values with the corresponding EFSA breakpoints [65].

### 4.3. Whole-Genome Sequencing, Assembly, and Annotation

#### 4.3.1. DNA Extraction, Library Preparation, and Whole-Genome Sequencing

Genomic DNA of *Lactiplantibacillus plantarum* L3 was extracted using the Novogene DNA isolation and purification kit, according to the manufacturer’s instructions. DNA quality and quantity were assessed as part of the standard quality-control procedures prior to library preparation in Novogene, UK.

Whole-genome sequencing libraries were prepared using the Novogene NGS DNA Library Prep Set (Cat. No. PT004). Sequencing was performed by Novogene using the Illumina NovaSeq 6000 platform, generating paired-end reads of 150 bp (PE150).

Raw sequencing data were processed using Novogene’s standard quality-control pipeline. Low-quality reads, adapter sequences, and potential contaminants were removed before downstream analyses. High-quality reads were provided in FASTQ format and used for genome assembly and subsequent bioinformatic analyses.

#### 4.3.2. Genome Assembly and Identification

Sequencing reads were assembled using the Unicycler assembler with default parameters [66]. All contigs above 200 bp were included in this assembly. The whole-genome shotgun (WGS) project is available under NCBI accession NZ_JAZFNE020000000 in the NCBI database, and the genome assembly under accession GCF_036353295.2. Plasmid and chromosomal contigs were distinguished using the PlasmidFinder tool [67].

Species identification of the strain under study was performed using Average Nucleotide Identity (ANI) analysis. The whole-genome sequence of the isolate was compared with reference genomes representing closely related species within the *Lactiplantibacillus plantarum* group obtained from the GenBank database. Pairwise genome comparisons were conducted using the OrthoANIu algorithm, which calculates ANI values based on orthologous genomic regions shared between two genomes.

In addition to ANI values, the average aligned length and genome coverage percentages for both query and reference genomes were determined to assess the extent of genomic similarity. Species assignment was based on the widely accepted ANI threshold of 95–96%, above which strains are considered to belong to the same species. The obtained ANI values and alignment statistics were subsequently used to evaluate the taxonomic position of the studied isolate relative to the selected reference genomes.

Genome completeness and contamination were evaluated using CheckM2 (v. 1.1.0), a machine-learning–based tool for genome quality assessment [68]. The analysis was performed using default settings, which infer completeness and contamination from genomic feature patterns rather than relying solely on marker gene sets. CheckM2 provided genome-wide estimates of assembly completeness and potential contamination and was used in conjunction with BUSCO (v. 5.8.0) to obtain a comprehensive assessment of genome quality [69].

#### 4.3.3. Genome Visualization

- Reference-guided scaffolding and pseudo-chromosome construction

The draft genome assembly was scaffolded against the *Lactiplantibacillus plantarum* strain WCFS1 reference genome (NCBI Ref. Seq.: GCF_000203855.3) using RagTag v2.1.0 with default minimap2 alignment parameters. The resulting AGP file (ragtag.scaffold.agp) was filtered to retain only assembled sequence entries (“W”). Contigs that mapped uniquely and consistently to the chromosome or plasmids (p1, p2) were kept while unplaced or ambiguous contigs were excluded. Cumulative offsets derived from the AGP file were used to convert all feature coordinates from contig relative to continuous pseudo-chromosome coordinates.

- Feature Extraction and Derived Input Tables

CDS features were extracted from the genome annotation (GFF3) and converted to BED format (GCF_contig_CDS.bed). GC content and GC skew were computed in non-overlapping 5 kb windows for each contig, producing GCF_contig_gc_content.tsv and GCF_contig_gc_skew.tsv. These tables were merged with AGP-derived offsets to obtain continuous coordinates. Gene density was calculated within a custom R script as the number of CDS per 5 kb window. When COG annotations were unavailable, CDS were assigned fallback forward/reverse categories for strand-based coloring.

- GC content, GC skew, and *oriC* inference

GC content was calculated as (G + C)/(A + T + G + C), and GC skew as (G − C)/(G + C). GC skew was plotted with a zero baseline to highlight replication-associated asymmetry. The putative origin of replication (oriC) was inferred from the position of the *dnaA* locus and the local inversion point of GC skew along the pseudo-chromosome. The coordinate was converted to continuous genome coordinates using AGP-derived contig offsets and plotted as a point marker.

- Circular genome visualization

Circular genome plots were generated in R using circlize v0.4.16. Tracks (outer → inner) included: 1. 100 kb labels and 10/100 kb tick marks; 2. Replicon blocks (chromosomes vs. plasmids); 3. Gene density heatmap; 4. CDS orientation arrows; 5. GC content; 6. GC skew; 7. *oriC* marker.

All tracks were drawn in the contig order defined by the RagTag AGP file. Replicon labels were added manually during figure preparation.

#### 4.3.4. Genome Annotation

The assembled genome was annotated with the universal prokaryotic annotator Prokka v1.14.5 (Seemann, 2014), an automated genome annotation pipeline that uses its integrated protein database [70].

Functional annotation was further performed using the BlastKOALA online tool [71]. Predicted protein sequences (FAA files) generated by Prokka were used as input. Proteins were assigned to Kyoto Encyclopedia of Genes and Genomes (KEGG) Orthology (KO) groups and subsequently categorized into functional pathways to facilitate comparative analysis of the genome.

#### 4.3.5. Functional Annotation

Functional annotation was performed using BLASTp (NCBI BLAST+ v2.2.28) by comparing predicted protein sequences generated by Prokka against the *Lactiplantibacillus plantarum* WCFS1 reference proteome. Genes of interest were identified through a targeted genome-mining approach. Candidate genes were selected based on previously published literature describing stress-associated (acid/bile tolerance, etc.) determinants in lactic acid bacteria and were subsequently searched within the Prokka-annotated genome. Predicted protein sequences corresponding to the identified genes were further compared against reference sequences from *L. plantarum* WCFS1 using BLASTp to confirm gene identity and sequence conservation.

Prediction of bacteriocin gene clusters was performed using the BAGEL 4 webserver [72].

Putative antimicrobial resistance (AMR) genes were identified using the Comprehensive Antibiotic Resistance Database (CARD) and the Resistance Gene Identifier (RGI) tool, covering both chromosome and plasmid contigs.

### 4.4. Statistical Analysis

All in vitro experiments were performed in triplicate (n = 3), and the results are presented as mean ± standard deviation (SD). Data normality was assessed using the Shapiro–Wilk test. Based on the normality results, statistical differences among groups were analyzed using one-way analysis of variance (ANOVA) followed by Tukey’s post hoc test. All statistical analyses were performed using GraphPad Prism 8 (GraphPad Software, San Diego, CA, USA). Differences were considered statistically significant at *p* < 0.05. Statistical significance is indicated in tables and figures as follows: *p* < 0.05 *, *p* < 0.01 **, and *p* < 0.001 ***.

## 5. Conclusions

This study provides a comprehensive phenotypic and genomic characterization of *strain* L3, isolated from the traditional fermented dairy product katak. Whole-genome sequencing and ANI analysis confirmed its taxonomic identity as *L. plantarum*.

In vitro phenotypic analyses demonstrated strain-specific antioxidant, antibacterial, and antifungal activities against a range of undesirable microorganisms, highlighting its antimicrobial potential and free-radical scavenging activity in both DPPH and ABTS assays. Genome analysis revealed features commonly associated with *L. plantarum*, including genes involved in carbohydrate metabolism, environmental adaptation, and stress response. Genes previously associated with acid, bile, and oxidative stress tolerance were identified, along with a plantaricin-associated gene cluster containing structural, regulatory, transport, and immunity-related genes. These findings suggest the genetic potential for adaptation to diverse environmental conditions and for bacteriocin production. Importantly, no acquired or potentially transferable antimicrobial resistance determinants were detected, supporting the favorable genomic safety profile of strain L3.

Taken together, the phenotypic and genomic data identify *L. plantarum* L3 as a candidate with probiotic-associated traits, as indicated by in vitro tests. However, the genomic predictions reported in this study do not demonstrate gene expression or functionality under specific conditions. Therefore, additional transcriptomic, proteomic, and in vivo studies are required to validate the biological significance of the identified genetic traits.

## Figures and Tables

**Table 1:** OrthoANI-based genome comparison between the L3 studied strain and reference genomes.

Reference Species	Genome Assembly	OrthoANI Identity (%)	Average Aligned Length (bp)	Query Genome Coverage (%)	Reference Genome Coverage (%)	Taxonomic Interpretation
*Lactiplantibacillus plantarum* ASM991365v1	GCF_009913655.1	99.07	2,062,387	64.23	63.99	Same species
*Lactiplantibacillus plantarum* WCFS1	GCF_000203855.3	98.04	2,129,922	66.33	63.64	Same species
*Lactiplantibacillus paraplantarum* ASM143565v1	GCF_001435655.1	86.47	1,596,083	49.71	48.60	Distinct species
*Lactiplantibacillus pentosus* ASM5374859v1	GCA_053748595.1	80.41	1,423,999	44.35	39.66	Distinct species

**Table 2:** Antimicrobial activity of postbiotics and cell-free supernatants derived from strain L3 against selected pathogens determined by agar well diffusion assay.

Test Pathogens	Tested Samples	Inhibition Zone Diameter (mm ± SD) *
*Staphylococcus aureus* V00037	Cells (48 h, MRS)	7.83 ± 0.29 ^b^
	aCFS (24 h, MRS)	4.67 ± 0.58 ^c^
	aCFS (48 h, MRS)	4.17 ± 0.29 ^c^
	Soy milk	4.08 ± 0.14 ^c^
	10% skimmed milk	-
	Erythromycin (positive control)	17.00 ± 0.00 ^a^
*Escherichia coli* ATCC 25922	aCFS (24 h, MRS)	6.33 ± 0.29 ^d^
	aCFS (48 h, MRS)	14.75 ± 0.25 ^b^
	nCFS (48 h, MRS)	14.17 ± 0.29 ^b^
	Soy milk (24 h)	4.33 ± 0.29 ^e^
	10% skimmed milk	5.00 ± 0.00 ^e^
	Chloramphenicol (positive control)	18.50 ± 0.50 ^a^
	Ciprofloxacin (positive control)	9.67 ± 0.58 ^c^
*Escherichia coli* HB101	aCFS (24 h, MRS)	11.83 ± 0.29 ^a^
	aCFS (48 h, MRS)	10.00 ± 0.00 ^b^
	Heat-treated aCFS (70 °C, 10 min)	9.33 ± 0.58 ^b^
	aCFS (48 h) + proteinase K	6.83 ± 0.29 ^c^
	Soy milk (24 h)	0.00 ± 0.00 ^d^
	10% skimmed milk	-
	3.3% acetic acid (pH 4.75)	3.17 ± 0.29 ^d^
	Ciprofloxacin (positive control)	34.67 ± 2.12
*Pseudomonas aeruginosa* PAO1	Cells (48 h, MRS)	4.83 ± 0.29 ^a^
	aCFS (48 h, MRS)	4.18 ± 0.32 ^b^
	10% skimmed milk	0.00 ± 0.00 ^c^
	Soy milk	-
	Ciprofloxacin (positive control)	34.83 ± 0.29
*Chromobacterium violaceum* DSMZ 30191	aCFS (24 h, MRS)	5.17 ± 0.29 ^a^

* Values are expressed as mean ± standard deviation (SD) of three independent replicates (*n* = 3). Different superscript letters within each pathogen group indicate statistically significant differences among treatments according to one-way ANOVA followed by Tukey’s multiple comparisons test (*p* < 0.05). “-” indicates not tested. Abbreviations: aCFS—acidic cell-free supernatant; nCFS—neutralized cell-free supernatant.

**Table 3:** Results of the GC-MS metabolomics analysis of produced metabolites during the fermentation of *L. plantarum* L3 in MRS broth (pH 6.5).

Metabolites, % of TIC	Control MRS	CFS, 24 h	CFS, 48 h
Butyric acid	0.13	0.17	0.25
Ethylene glycol	0.19	0.25	0.39
Propan-1,2-diol	0.21	0.29	0.57
Pyruvic acid	2.56	3.46	0.80
Lactic acid	16.03	21.64	27.45
β-Phenyllactic acid	0.14	0.21	0.32
Oxalic acid	1.21	1.63	0.93

TIC—Total Ion Current.

**Table 4:** Minimum inhibitory concentrations (MICs) of antibiotics tested against strain L3, interpreted according to EFSA cut-off values for probiotic safety.

Antibiotic	Results (µg)	EFSA Cut-Off	R/S
Chloramphenicol	1.5	8	S
Tetracycline	1.5	32	S
Streptomycin	32	64	S
Erythromycin	0.38	1	S
Clindamycin	0.19	4	S
Gentamicin	1.5	16	S
Vancomycin	R	n.r.	R
Kanamycin	24	64	S

Legend: Susceptible (S): inhibited at concentrations ≤ the EFSA breakpoint (S ≤ x mg/L). Resistant (R): not inhibited at concentrations > the EFSA breakpoint (R > x mg/L); n.r., not required due to intrinsic resistance.

**Table 5:** Assembly statistics of the draft genome of strain L3.

Parameter	Value
Reads generated	7,145,069
Assembly size (bp)	3,260,383
GC content (%)	44.5
Total contigs	119
Chromosomal contigs	98
Predicted CDSs	3109
tRNAs	64
rRNAs	2
N50 (bp)	142,766
L50	8
Coverage	546
Completeness BUSCO (%)	98.3
Completeness Check M2 (%)	99.02
contamination	0.50%

**Table 6:** Genes potentially associated with stress-adaptation in strain L3.

Function	Genes
Acid and bile tolerance	*rpsS*; *ppk*; *ackA1/2*; *fabH1/2*; *pepF1*; *arcB*; *copA*; *pgk*; *dnaK*; *dnaJ*; *groS*; *grpE*; *htrA*
Acid stress	*atpA–H*; *clpB*; *clpP1/2*; *dltC1/2*; *dltD*; *gap*; *groL*; *ldh1–3*; *pgi*; *plsC*; *pyk*; *recA*; *relA*; *tpiA*; *yjbM*; *ywaC*; *nhaC1/2*; *lepA1/2*
Bile resistance	*argS*; *dps1/2*; *glf1–3*; *glnA*; *nagB*; *oppA1–3*; *pyrG*; *ppaC*; *cfa1/2*; *cbh*; *celB*
Oxidative stress	*fnr1/2*; *nrdH*; *mntH1–4*; *mntB1/2*; *npr1–3*; *trxA1–3*; *trxB*; *msrA1–3*; *msrC*; *tpx*
Biofilm formation	*luxS*, *comC*, *comEA*, *comEC*

**Table 7:** Putative antimicrobial resistance genes identified in the genome of *L. plantarum* L3 using CARD/RGI.

Predicted AMR Gene	Resistance Class	Mechanism	Identity (%)	Model Type	Associated Compound
*vanY*	Glycopeptide antibiotics	Antibiotic target alteration	31.93	Protein homolog model	Vancomycin
*qacJ*	Disinfecting agents and antiseptics	Antibiotic efflux	40.2	Protein homolog model	Benzalkonium chloride

## Data Availability

The original contributions presented in this study are included in the article/Supplementary Material. Further inquiries can be directed to the corresponding author.

## Supplementary Materials

The following supporting information can be downloaded at: https://www.mdpi.com/article/10.3390/ijms27167088/s1.
