# Supplementary material for: Bridging In Vitro Functionality and Genomic Insights in Lactiplantibacillus plantarum L3 Isolated from Traditional Bulgarian Katak
Source: Int J Mol Sci. 2026 Aug 7;27(16):7088. doi: 10.3390/ijms27167088 (PMC13512861; doi:10.3390/ijms27167088)
Supplement: Supplementary file 1 [file ijms-27-07088-s001.zip › ijms-4348515-supplementary.pdf]

Supplementary Table S1. Stress tolerance-associated genes identified in *Lactiplantibacillus plantarum* strain L3, including locus tags, predicted gene products, and sequence identity percentages relative to *L. plantarum* WFC1.

Function	Genes	Locus tag	Predicted product	% Identity
Acid and bile tolerance	*rpsS*	JDGCCJPA_01459	30S ribosomal protein S19	100
	ppk	JDGCCJPA_02282	polyphosphate kinase	100
	ackA	JDGCCJPA_00779	acetate kinase	100
	ack2	JDGCCJPA_02449	acetate kinase	100
	fabH1	JDGCCJPA_01047	3-oxoacyl-acyl-carrier-protein synthase III	99.69
	fabH2	JDGCCJPA_01314	3-oxoacyl-acyl-carrier-protein synthase III	99.7
	pepF1	JDGCCJPA_01816	oligoendopeptidase F	99.5
	arcB	JDGCCJPA_02195	ornithine carbamoyltransferase	99.12
	copA	JDGCCJPA_01618	copper-exporting P-type ATPase	99.69
	eno2	JDGCCJPA_01172	enolase	100
	pgk	JDGCCJPA_01170	phosphoglycerate kinase	100
	dnaK	JDGCCJPA_00681	chaperone protein DnaK	100
	dnaJ	JDGCCJPA_00680	chaperone protein DnaJ	100
	groS	JDGCCJPA_01111	10 kDa chaperonin	100
	grpE	JDGCCJPA_00682	protein GrpE;	99.50
	htrA	JDGCCJPA_01871	serine protease HtrA	100
Acid stress	*atpA*	JDGCCJPA_00887	ATP synthase subunit alpha	99.8
	atpB	JDGCCJPA_00891	ATP synthase subunit a	99.58
	atpC	JDGCCJPA_00884	ATP synthase epsilon chain	100
	atpD	JDGCCJPA_00885	ATP synthase subunit beta	100
	atpE	JDGCCJPA_00890	ATP synthase subunit c	100
	atpF	JDGCCJPA_00889	ATP synthase subunit b	100
	atpG	JDGCCJPA_00886	ATP synthase gamma chain	100
	atpH	JDGCCJPA_00888	ATP synthase subunit delta	100
	clpB	JDGCCJPA_00588	chaperone protein ClpB	99.88
	clpP	JDGCCJPA_01165	ATP-dependent Clp protease proteolytic subunit	99.49
	dltC1	JDGCCJPA_00158	D-alanyl carrier protein	100
	dltC2	JDGCCJPA_00675	D-alanyl carrier protein;	100
	dltD	JDGCCJPA_00674	protein DltD	99.76
	gap3	JDGCCJPA_01169	glyceraldehyde-3-phosphate dehydrogenase	100
	groL	JDGCCJPA_01112	60 kDa chaperonin	100
	ldh1	JDGCCJPA_01510	L-lactate dehydrogenase	99.68
	ldh2	JDGCCJPA_01752	L-lactate dehydrogenase	100
	ldh3	JDGCCJPA_02191	L-lactate dehydrogenase	100
	pgi	JDGCCJPA_00324	glucose-6-phosphate isomerase	99.56
	plsC	JDGCCJPA_00712	1-acyl-sn-glycerol-3-phosphate acyltransferase	98.58

	pyk	JDGCCJPA_00583	pyruvate kinase	99.83
	recA	JDGCCJPA_00827	protein RecA	100
	relA	JDGCCJPA_00663	GTP pyrophosphokinase	99.87
	tpiA	JDGCCJPA_01171	triosephosphate isomerase	100
	yjbM	JDGCCJPA_01814	GTP pyrophosphokinase YjbM	100
	ywaC	JDGCCJPA_02849	GTP pyrophosphokinase YwaC	100
	nhaC1/nha1	JDGCCJPA_02082	Na+/H+ antiporter NhaC	99.57
	nhaC2/nha2	JDGCCJPA_02552	Na+/H+ antiporter NhaC	99.58
	lepA1	JDGCCJPA_00673	elongation factor 4	99.84
	lepA2	JDGCCJPA_01674	elongation factor 4	98.66
Bile resistance	*argS*	JDGCCJPA_00146	arginine–tRNA ligase	99.29
	dps1	JDGCCJPA_01681	DNA protection during starvation protein	100
	dps2	JDGCCJPA_02996	DNA protection during starvation protein	100
	glf1	JDGCCJPA_02690	UDP-galactopyranose mutase	98.67
	glf2	JDGCCJPA_03022	UDP-galactopyranose mutase	98.10
	glnA	JDGCCJPA_00314	glutamine synthetase	100
	nagB	JDGCCJPA_02603	glucosamine-6-phosphate deaminase	100
	oppA1	JDGCCJPA_01163	oligopeptide-binding protein OppA	99.82
	oppA2	JDGCCJPA_01891	oligopeptide-binding protein OppA	99.81
	oppA3	JDGCCJPA_02171	oligopeptide-binding protein OppA	98.35
	pyrG	JDGCCJPA_02222	CTP synthase	100
	ppaC	JDGCCJPA_02705	inorganic pyrophosphatase	100
	cfa1	JDGCCJPA_01293	cyclopropane-fatty-acyl-phospholipid synthase	98.97
	cfa2	JDGCCJPA_02781	cyclopropane-fatty-acyl-phospholipid synthase	99.50
	cbh	JDGCCJPA_03092	choloylglycine hydrolase	99
	celB	JDGCCJPA_02260	PTS cellobiose-specific EIIA component	99.78
Oxidative stress	*fnr1*	JDGCCJPA_00188	fumarate and nitrate reduction regulatory protein	99.53
	fnr2	JDGCCJPA_02740	fumarate and nitrate reduction regulatory protein	98.64
	nrdH	JDGCCJPA_01084	glutaredoxin-like protein NrdH	100
	mntH1	JDGCCJPA_00020	divalent metal cation transporter MntH	100

	mntH2	JDGCCJPA_01577	divalent metal cation transporter MntH	100
	mntH3	JDGCCJPA_02645	divalent metal cation transporter MntH	99.78
	mntB1	JDGCCJPA_01506	manganese transport ATP-binding protein MntB	99.59
	mntB2	JDGCCJPA_01507	manganese transport ATP-binding protein MntB	99.66
	npr1	JDGCCJPA_00191	NADH peroxidase	99.32
	npr2	JDGCCJPA_00359	NADH peroxidase	100
	trxA1	JDGCCJPA_00805	thioredoxin	100
	trxA2	JDGCCJPA_02611	thioredoxin	100
	trxA3	JDGCCJPA_02735	thioredoxin	99.06
	trxB	JDGCCJPA_01145	thioredoxin reductase	100
	msrA1	JDGCCJPA_00055	peptide methionine sulfoxide reductase MsrA	100
	msrA2	JDGCCJPA_00656	peptide methionine sulfoxide reductase MsrA	99.43
	msrA3	JDGCCJPA_02703	peptide methionine sulfoxide reductase MsrA	100
	msrB	JDGCCJPA_02704	peptide methionine sulfoxide reductase MsrB	100
	msrC	JDGCCJPA_00850	free methionine-R-sulfoxide reductase	100
	tpx	JDGCCJPA_00845	thiol peroxidase	99.39
Biofilm formation	*luxS*	JDGCCJPA_01156	S-ribosylhomocysteine lyase	100
	comC	JDGCCJPA_01450	type IV prepilin-like leader peptidase	99.12
	comEA	JDGCCJPA_01738	ComE operon protein 1	99.14
	comEC	JDGCCJPA_01736	ComE operon protein 3	99.44
